# Supplementary material for: Oxidative stress-driven enhanced iron production and scavenging through Ferroportin reorientation worsens anemia in antimony-resistant Leishmania donovani infection
Source: PLoS Pathog. 2025 Jan 31;21(1):e1012858. doi: 10.1371/journal.ppat.1012858 (PMC11785346; doi:10.1371/journal.ppat.1012858)

**S1 Table.**

**Table A:** Table showing the list of Glycolytic genes presented in the heatmap of Fig 2E.i. with their respective enrichment scores.


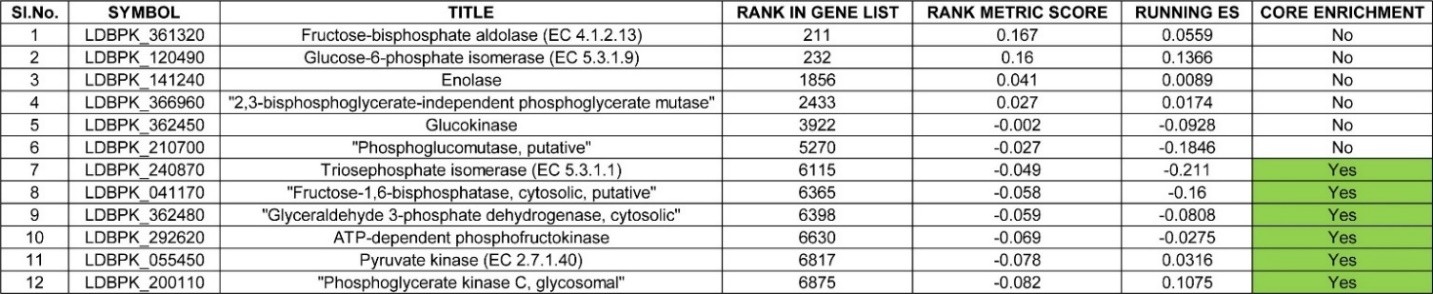


**Table B:** Table showing the list of Pentose phosphate pathway (PPP) genes presented in the heatmap of Fig 2E.ii. with their respective enrichment scores.


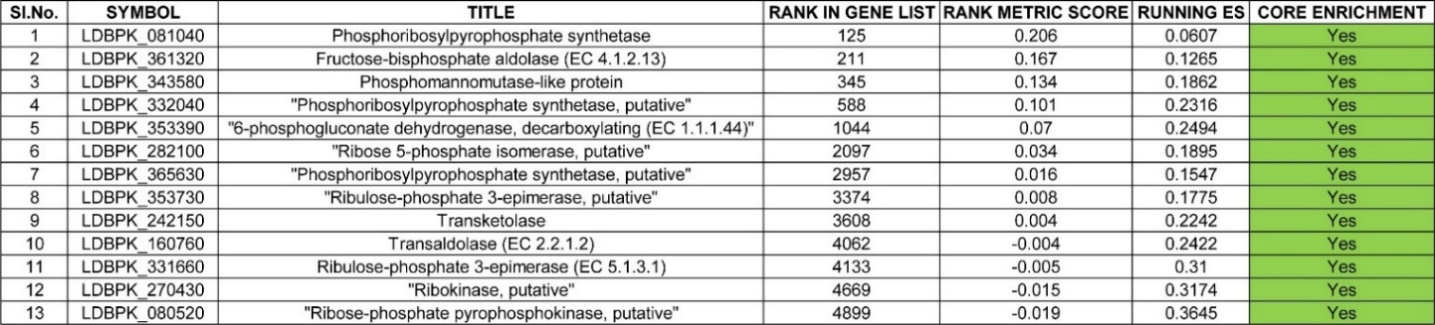

Supplement: S1 Table — (A) Table showing the list of Glycolytic genes presented in the heatmap of Fig 2E.i with their respective enrichment scores. (B) Table showing the list of Pentose phosphate pathway (PPP) genes presented in the heatmap of Fig 2E.ii with their respective enrichment scores. (DOCX) [file ppat.1012858.s001.docx]
