# Supplementary figures and images for: Oxidative stress-driven enhanced iron production and scavenging through Ferroportin reorientation worsens anemia in antimony-resistant Leishmania donovani infection
Source: PLoS Pathog. 2025 Jan 31;21(1):e1012858. doi: 10.1371/journal.ppat.1012858 (PMC11785346; doi:10.1371/journal.ppat.1012858)

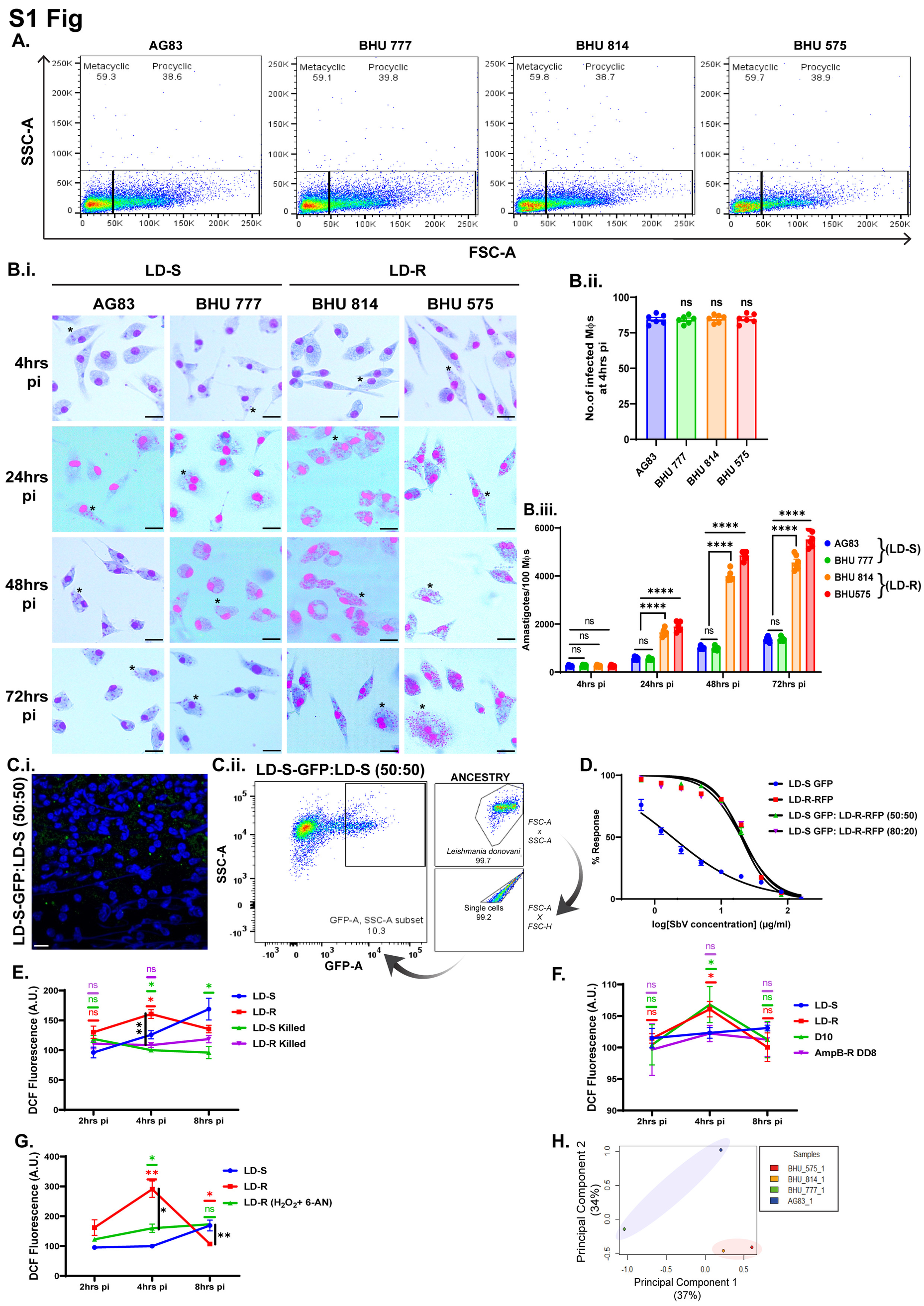

Supplement: S1 Fig — (A.) Representative flow cytometer images (BD LSRFortessaTM Cell Analyzer) showing the % of metacyclic promastigote in AG83, BHU777 (7th-day culture); BHU814, BHU575 (5th-day culture) after uniform sorting in Beckman Coulter Cytoflex srt. FSClow (left) represents the metacyclic population and FSChigh (right) represents the procyclic population. (B.i.) Giemsa-stained images of an equal number of AG83, BHU777, BHU814, and BHU575 metacyclic promastigote infected-MФs at 4 hrs, 24 hrs, 48 hrs, and 72 hrs pi. One representative small nucleus of LD has been marked in (*) to show the infected-MФs. (B.ii.) No. of infected MФs/100 MФs at 4 hrs pi were enumerated. Each data is the mean of three individual sets. (B.iii.) Amastigotes/100 MФs at 4 hrs, 24 hrs, 48 hrs, and 72 hrs pi were calculated taking different fields, and the data represents the SEM of three independent experiments. (C.i.) Confocal images of macerated spleen sample of LD-S-GFP: LD-S showing the GFP-amastigote load. Scale bars indicate 20 µm. (C.ii.) % of the GFP-positive population was enumerated from FACS to denote the load of LD-S-GFP. The right-most panel shows the ancestry of each analysis. (D.) The dose-response curve of clonal LD lines derived from LD-S-GFP, LD-R, LD-S-GFP: LD-R-RFP (50:50), and LD-S-GFP: LD-R-RFP (80:20) infected-spleen to SbV as determined from intracellular amastigotes/100 MФs for calculating EC50. (E.) DCF-fluorescence quantification of LD-S, LD-R, heat-killed-LD-S, and heat-killed-LD-R infected-MФs at early hours. Heat-killed LD-R experimental sets at 4 hrs pi significantly fail to generate ROS as compared to LD-R infected-MФs at 4hrs pi (**, P ≤ 0.01). (F.) DCF-fluorescence quantification of MФs infected with LD-S, LD-R, D10, and AmpB-R DD8 (G.) DCF-fluorescence quantification of MФs infected with LD-S or LD-R, or NADPHexh-LD-R by H2O2+6-AN treatment. LD-R (H2O2+6-AN) significantly failed to generate ROS as compared to LD-R infected-MФs at 4hrs pi (*, P ≤ 0.05). At 8hrs, LD-R infe [file ppat.1012858.s002.tif]

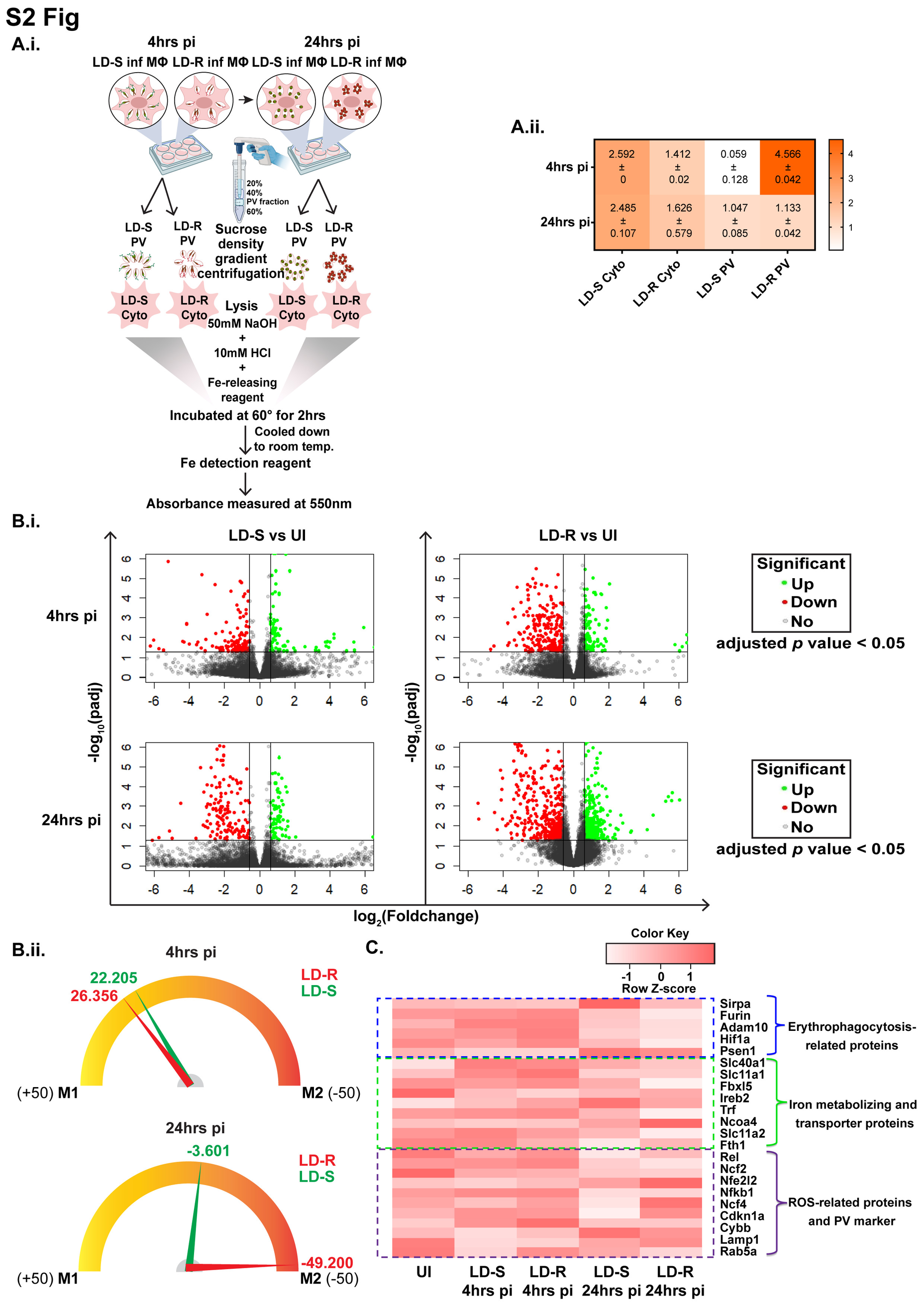

Supplement: S2 Fig — (A.i.) Scheme showing PV extraction from LD-S and LD-R infected-MФs at 4 hrs and 24 hrs pi ensued by quantification of iron following Ferrozine-based colorimetric assay. (A.ii.) Heatmap showing iron concentration in cytoplasmic-fraction and PV-fraction of LD-S and LD-R infected-MФs at 4 hrs and 24 hrs pi. Data are represented as Mean ± SEM. (B.i.) Volcano plots of differential gene expression of LD-S (left) and LD-R infected-MФs (right) versus uninfected control at 4 hrs pi (upper panel) and 24 hrs pi (lower panel). Genes above the significance threshold (adjusted p-value<0.05) are marked in green having log2(fold change) >0.6, i.e., upregulated, and red having log2(fold change) <−0.6, i.e., downregulated, while the rest are marked in grey. (B.ii.) Scheme representing macrophage polarization states (M1 on left and M2 on right) based on the differential gene expression of LD-S (green) and LD-R (red) infected-MФs at 4 hrs and 24 hrs pi. Score of M1/M2 gene cluster = Sum of respective M1/M2 log2(fold change) of genes with adjusted p-value < 0.05. The determinant score depicting the total polarization outcome is calculated as = Score of M1 gene cluster- Score of M2 gene cluster. At 4 hrs pi, the total score of genes contributing towards M1 polarization in the case of LD-S infection is −0.909 and LD-R infection is 16.237, while the total score of genes contributing towards M2 polarization in the case of LD-S infection is −23.115 and LD-R infection is −10.1189. At 24 hrs pi, the total score of genes contributing towards M1 polarization in the case of LD-S infection is −17.987 and LD-R infection is −52.832, while the total score of genes contributing towards M2 polarization in the case of LD-S infection is −14.385 and LD-R infection is 3.631. (C.) Heatmap showing the differential expression pattern of erythophagocytosis-related protein (demarcated in blue), iron-metabolizing and transporter protein (green), and ROS-related proteins (purple) in uninfected-MФs, LD-S, and LD- [file ppat.1012858.s003.tif]

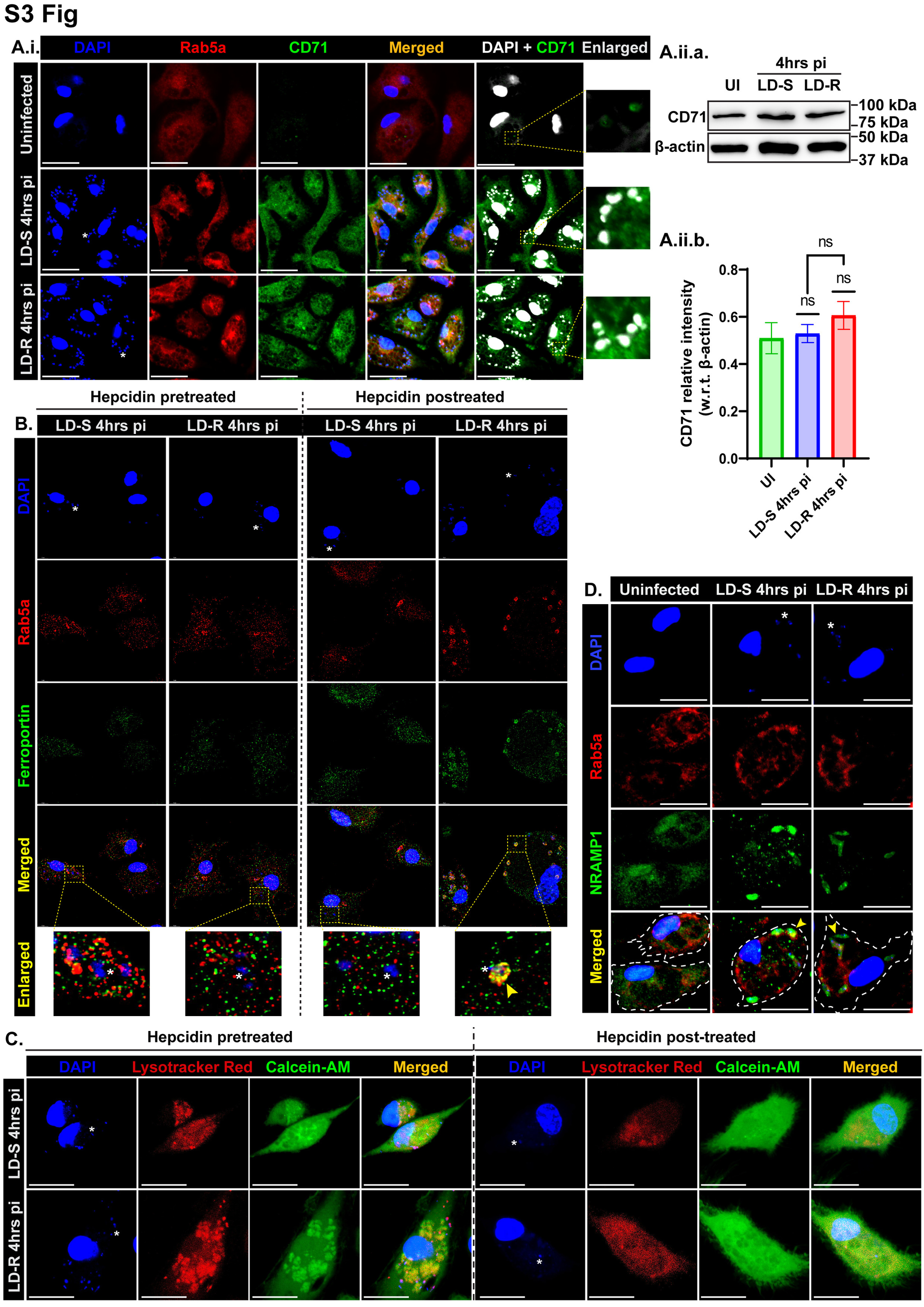

Supplement: S3 Fig — Confocal images showing the status of CD71 expression (green) in LD-S (upper panel) and LD-R-infected-MФs (lower panel) at 4 hrs pi. Rab5a (red) demarcates early PV. (A.ii.a.) Western blot showing expression of CD71 in the whole cell lysate of uninfected-MФs, LD-S, and LD-R infected-MФs at 4 hrs pi. (A.ii.b.) Relative intensity of CD71 western blot, i.e., fold change with respect to β-actin where no significant change in the relative intensity of CD71 in UI, LD-S 4hrs pi, and LD-R 4 hrs pi (P > 0.05) is observed. (B.) The relative intensity of Ferroportin (Fpn) fold change with respect to β-actin showed significant upregulation of Fpn in both LD-S and LD-R 4 hrs pi (***, P ≤ 0.001) as compared to UI control, with significant upregulation in LD-R 4 hrs pi as compared to LD-S 4hrs pi (***, P ≤ 0.001). (C.) Entire panels of Super-resolution images of hepcidin pretreated and hepcidin post-treated sets from Fig 4D.i showing Ferroportin localization. The lowermost panel shows the enlarged view of a portion showing Ferroportin localization with the Rab5a and LD nucleus (small blue dot) (D.) Entire panels of live cell confocal images showing iron status by staining with Calcein-AM (green) with lysotracker Red that demarcates PV in hepcidin pretreatment (left 4 panels) and hepcidin post-treatment (right 4 panels) experimental sets (separated by dotted line) elaborated from Fig 4D.ii (E.) Entire panels of confocal images of NRAMP1 expression by labeling with anti-NRAMP1 (green) and anti-Rab5a (red) antibodies elaborated from Fig 4E.i.a The dotted area in the merged panel demarcates the MФ boundary. Yellow arrows show one representative LD-PV with NRAMP1. (F.) The relative intensity of NRAMP1, i.e., fold change with respect to β-actin showing significant downregulation of NRAMP1 in both LD-S and LD-R at 4 hrs pi as compared to UI, whereas no significant change is observed in between both these infected experimental conditions. Each densitometry analysis is represented as a bar [file ppat.1012858.s004.tif]

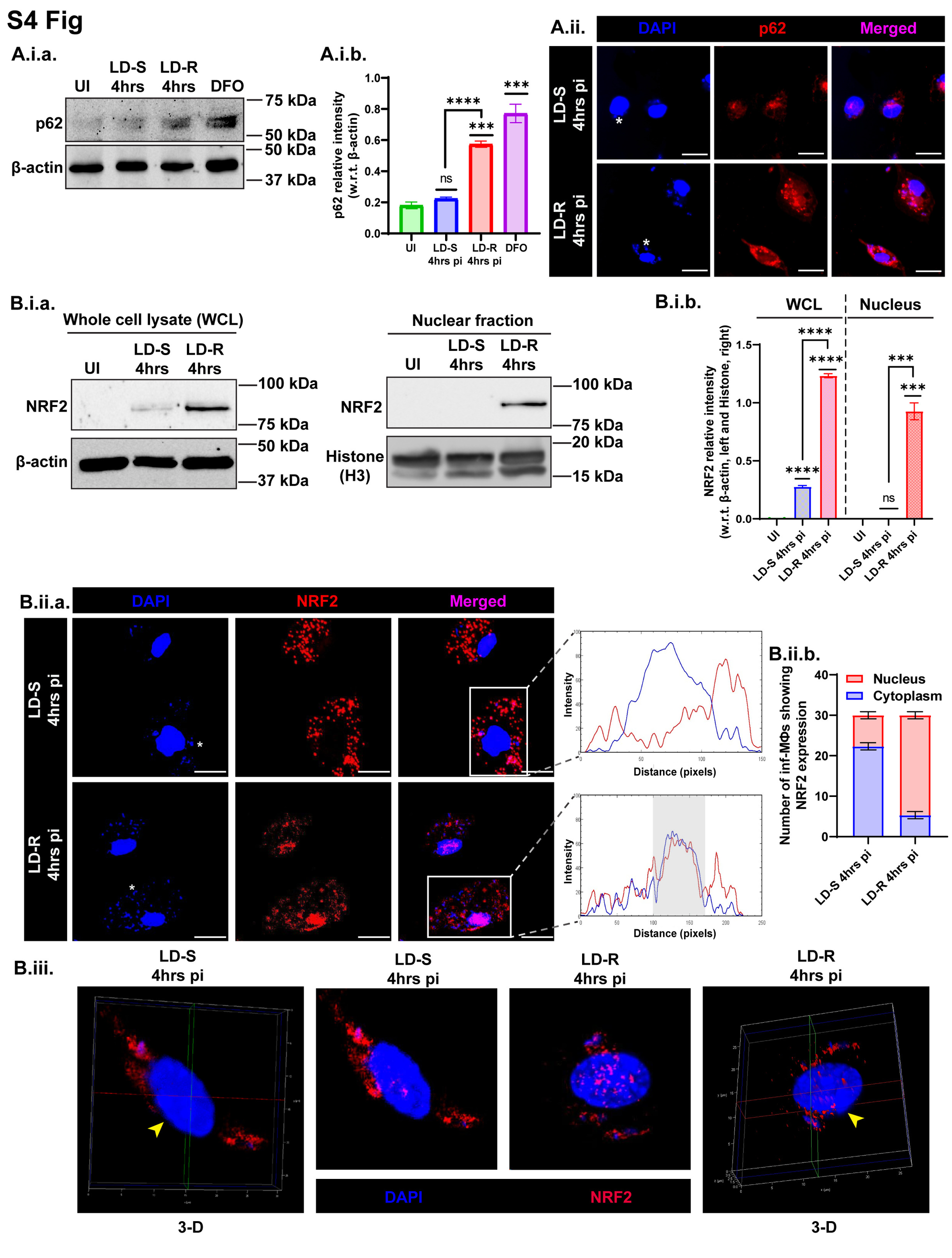

Supplement: S4 Fig — Western blot of whole cell lysate showing the expression of p62 in uninfected-MФs, LD-S, LD-R infected-MФs at 4 hrs pi and DFO-treated MФs keeping β-actin as the loading control. (A.i.b.) Relative intensity of p62 with respect to β-actin showed a significant upregulation of p62 in both LD-R 4 hrs pi and DFO-treated control as compared to UI (***, P ≤ 0.001). Also, significant upregulation in p62 expression in LD-R 4 hrs pi as compared to LD-S 4hrs pi (****, P ≤ 0.0001) is observed. (A.ii.) Confocal images showing the expression pattern of p62 (red) in LD-S and LD-R infected-MФs at 4 hrs pi. (B.i.a.) Western blot of whole cell lysate (left panel) and nuclear fraction (right panel) of uninfected-MФs, LD-S, and LD-R infected-MФs at 4 hrs pi showing the expression level of NRF2 keeping β-actin and Histone (H3) as loading control respectively. (B.i.b.) The relative intensity of NRF2 from whole cell lysate (left panel) with respect to β-actin showed significant upregulation of NRF2 in both LD-S and LD-R infected MФs 4 hrs pi (****, P ≤ 0.0001). Also, there is significant upregulation in LD-R 4 hrs pi vs LD-S 4hrs pi (****, P ≤ 0.0001), significant upregulation of nuclear-NRF2 (right panel) in LD-R 4 hrs pi as compared to both UI and LD-S 4hrs pi (***, P ≤ 0.001) is observed. No significant change was observed in the nuclear translocation of NRF2 with respect to uninfected control for LD-S infection. Each densitometry analysis is represented as a bar graph of Mean ± SEM for 3 biological replicates. (B.ii.a.) Confocal images showing the localization of NRF2 in LD-S and LD-R infected-MФs at 4 hrs pi. The left panel shows the RGB-profile plot of NRF2 (red) and DAPI (blue) where the X-axis denotes distance in pixels and the Y-axis denotes intensity. The grey area in the RGB-profile plot shows the colocalized region of NRF2 with the nucleus. (B.ii.b.) Stacked bar graphs representing the number of infected macrophages showing nuclear and cytoplasmic NRF2 among LD-S and LD-R-infe [file ppat.1012858.s005.tif]
