# Supplementary material for: Oxidative stress-driven enhanced iron production and scavenging through Ferroportin reorientation worsens anemia in antimony-resistant Leishmania donovani infection
Source: PLoS Pathog. 2025 Jan 31;21(1):e1012858. doi: 10.1371/journal.ppat.1012858 (PMC11785346; doi:10.1371/journal.ppat.1012858)
Supplement: S1 Video — Videography (20 frames/sec) showing active invasion of LD-S-GFP and LD-R-RFP metacyclic promastigotes in peritoneal macrophages at 4 hrs pi (left panel). The right panel shows LD-R-RFP amastigotes outcompeting LD-S-GFP amastigotes at 24 hrs pi. (PPTX) [file ppat.1012858.s006.pptx]

## Slide 1
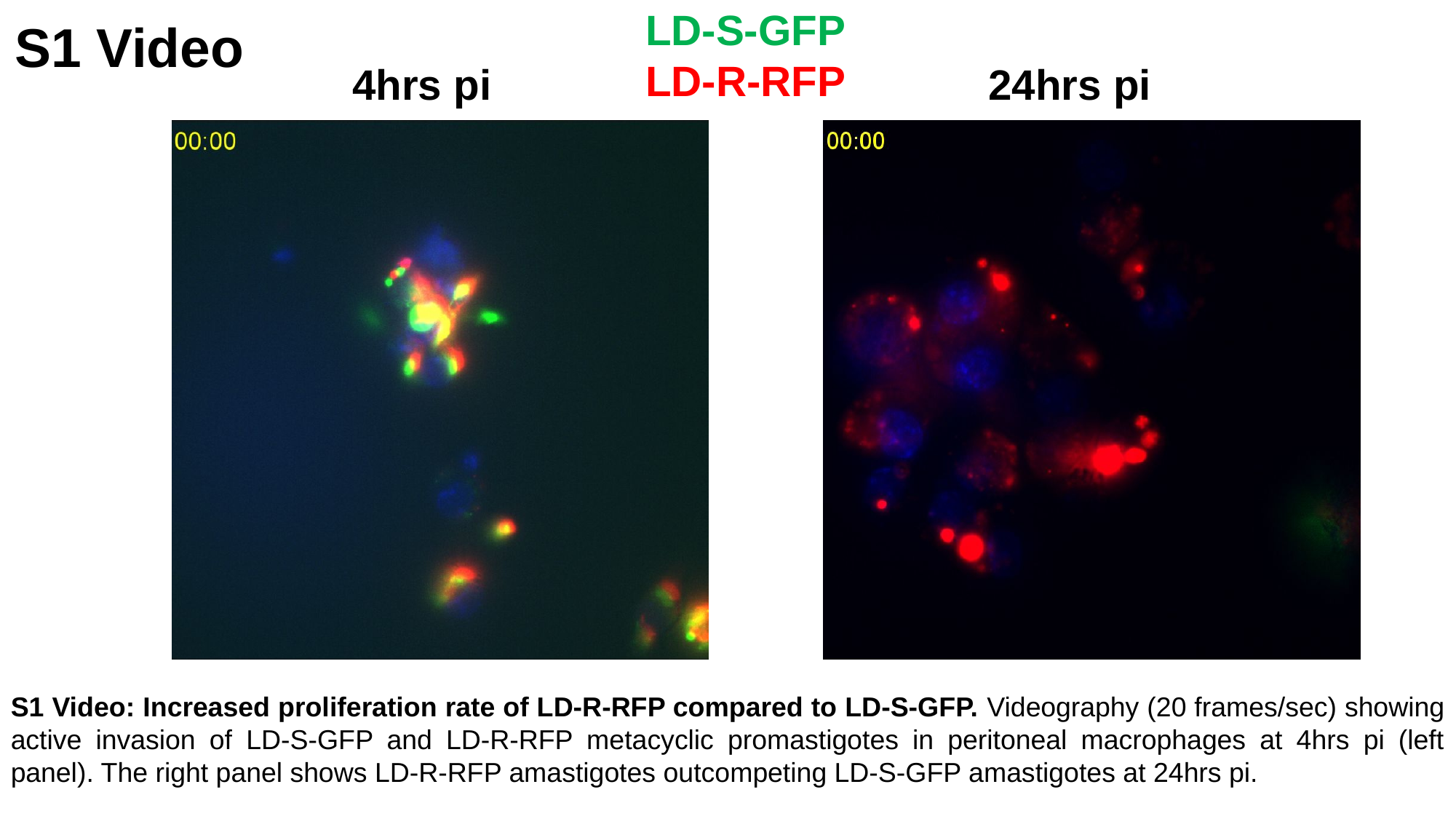

LD-S-GFP
LD-R-RFP
S1 Video
4hrs pi
24hrs pi
S1 Video: Increased proliferation rate of LD-R-RFP compared to LD-S-GFP. Videography (20 frames/sec) showing active invasion of LD-S-GFP and LD-R-RFP metacyclic promastigotes in peritoneal macrophages at 4hrs pi (left panel). The right panel shows LD-R-RFP amastigotes outcompeting LD-S-GFP amastigotes at 24hrs pi.
