# Supplementary material for: Oxidative stress-driven enhanced iron production and scavenging through Ferroportin reorientation worsens anemia in antimony-resistant Leishmania donovani infection
Source: PLoS Pathog. 2025 Jan 31;21(1):e1012858. doi: 10.1371/journal.ppat.1012858 (PMC11785346; doi:10.1371/journal.ppat.1012858)
Supplement: S2 Video — Videography (20 frames/sec) showing Ferroportin localized around macrophage surface in LD-S 4hrs pi (left panel) while it surrounds LD (small blue dot represents LD nucleus) in LD-R 4 hrs pi. (PPTX) [file ppat.1012858.s007.pptx]

## Slide 1
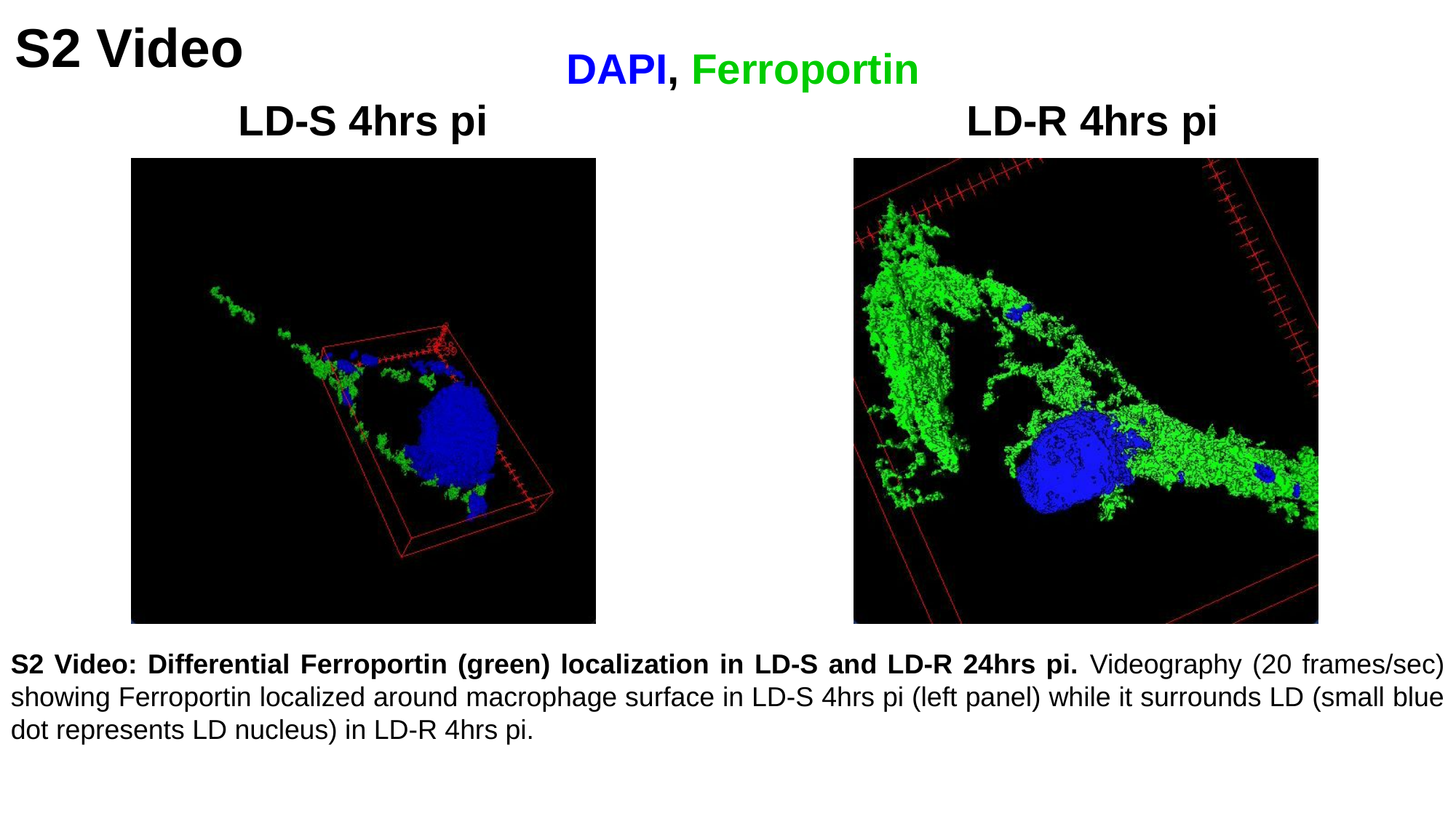

S2 Video
DAPI, Ferroportin
LD-S 4hrs pi
LD-R 4hrs pi
S2 Video: Differential Ferroportin (green) localization in LD-S and LD-R 24hrs pi. Videography (20 frames/sec) showing Ferroportin localized around macrophage surface in LD-S 4hrs pi (left panel) while it surrounds LD (small blue dot represents LD nucleus) in LD-R 4hrs pi.
