# Supplementary material for: Oxidative stress-driven enhanced iron production and scavenging through Ferroportin reorientation worsens anemia in antimony-resistant Leishmania donovani infection
Source: PLoS Pathog. 2025 Jan 31;21(1):e1012858. doi: 10.1371/journal.ppat.1012858 (PMC11785346; doi:10.1371/journal.ppat.1012858)
Supplement: S3 Video — Videography (20 frames/sec) showing Furin localization around perinuclear space in LD-S 24hrs pi (left panel) and colocalization with ADAM10 around membrane surface in LD-R 24 hrs pi (right panel) where CD11b (green) is a macrophage membrane raft marker. The right panel of each video represents a snapshot of the 3D Z-stack image with a white arrow demarcating the localization of Furin. (PPTX) [file ppat.1012858.s008.pptx]

## Slide 1
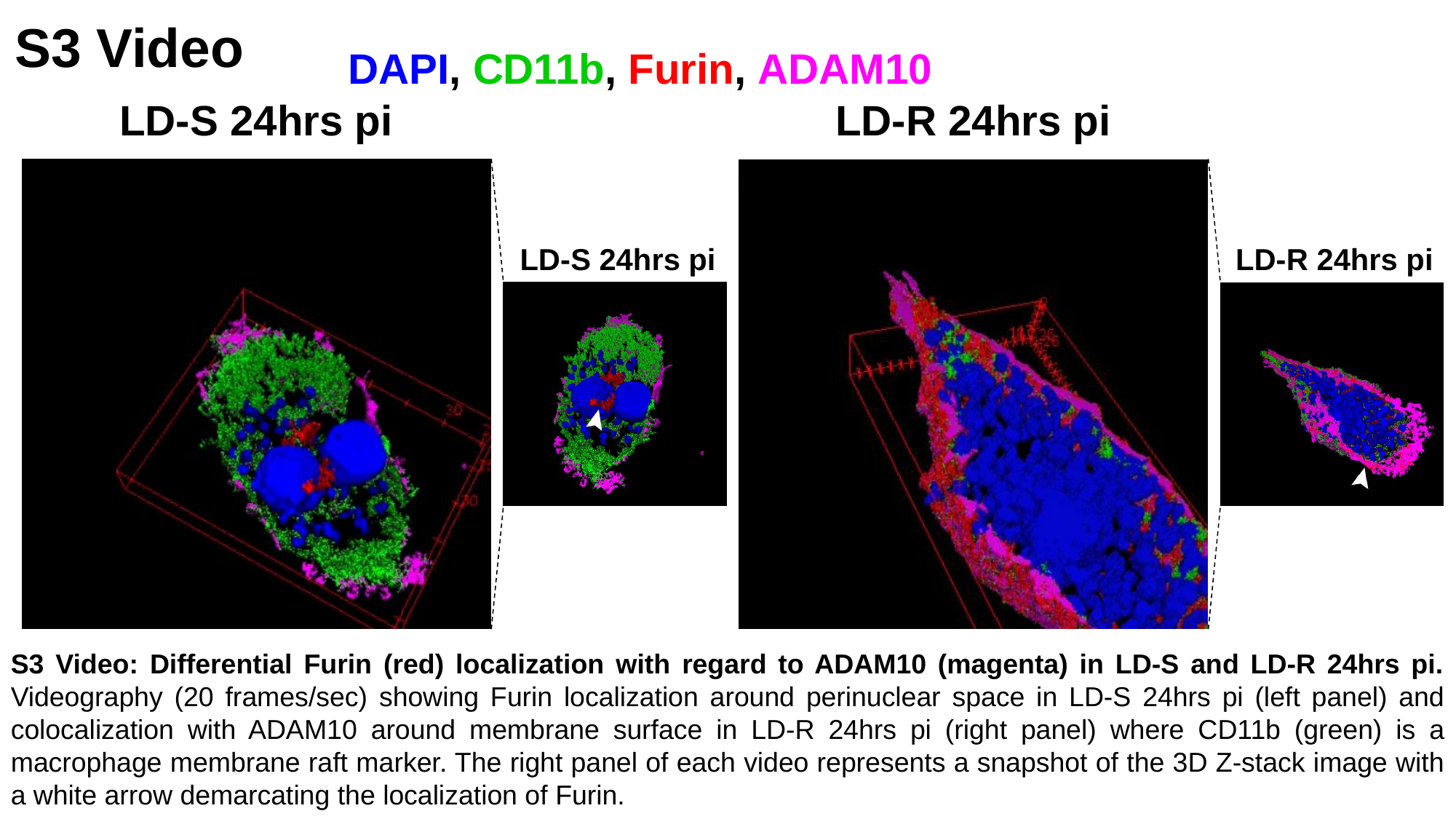

S3 Video
DAPI, CD11b, Furin, ADAM10
LD-S 24hrs pi
LD-R 24hrs pi
LD-S 24hrs pi
LD-R 24hrs pi
S3 Video: Differential Furin (red) localization with regard to ADAM10 (magenta) in LD-S and LD-R 24hrs pi. Videography (20 frames/sec) showing Furin localization around perinuclear space in LD-S 24hrs pi (left panel) and colocalization with ADAM10 around membrane surface in LD-R 24hrs pi (right panel) where CD11b (green) is a macrophage membrane raft marker. The right panel of each video represents a snapshot of the 3D Z-stack image with a white arrow demarcating the localization of Furin.
