# Supplementary material for: Oxidative stress-driven enhanced iron production and scavenging through Ferroportin reorientation worsens anemia in antimony-resistant Leishmania donovani infection
Source: PLoS Pathog. 2025 Jan 31;21(1):e1012858. doi: 10.1371/journal.ppat.1012858 (PMC11785346; doi:10.1371/journal.ppat.1012858)
Supplement: Attachment — Submitted filename: PPATHOGENS-D-24-00843_Authors Response.pdf [file ppat.1012858.s011.pdf]

To
The Editors
Plos Pathogens

Dear Editors

This is regarding your recent rejection letter on the submission of our paper to Plos Pathogens (**PPATHOGENS-D-24-00843**). Although every rejection is disappointing, we appreciate the time and effort the reviewers have given to provide their assessment of this manuscript. We would like to thank them all. We have carefully gone through the decision letter and we noted that the decision to reject the manuscript is primarily due to several concerns raised by Reviewer 1.

Broadly these concerns are:

A) Missing data: The "Missing data" that Reviewer 1 is referring to are already present in the manuscript (like data on organ parasite load), somehow the reviewer has missed them. Some other data that the reviewer pointed out to be missing like densitometry analysis of the western blots were already available with us, and we did not include them in the manuscript as it was only increasing the number of graphs and data volume without adding anything substantial. We are providing them with this rebuttal to make our point.

B) Inconsistent Methodology and Inadequate Statistical Analysis: We are providing evidence in this rebuttal to show that there were no major flaws in the methodology. Regarding statistical analysis, we have provided the P value along with its calculation which we believe will be able to remove any doubt on statistical analysis.

We are providing a point-by-point responses to every single concern raised by the reviewer and we are hoping that Editors will review these responses and reconsider their decision to reject our work. We strongly feel that there is only one additional experiment which Reviewer 1 has suggested will make the conclusion of this work stronger and we are prepared to conduct that. In view of these points, we appeal to allow us to submit a revision.

We look forward to hearing back from you.

Best Regards
Budhaditya

Reviewer #1: **The manuscript contains significant flaws that undermine the validity and reliability of its conclusions. The main issues are outlined below. Given these critical issues, the manuscript's conclusions are not substantiated by the data presented. The inconsistencies, lack of essential data, and inadequate methodological details significantly diminish the credibility and scientific contribution of this manuscript. Therefore, the manuscript is recommended for rejection.**

Response: We recognize that the reviewer has identified issues with our work. However, we believe that most of these comments do not raise significant concerns and are likely due to the reviewer either overlooking or misinterpreting our intended message, possibly due to the extensive amount of data. In the next paragraph, we have explained our reasoning and provided a detailed point-by-point response to all the concerns raised by the reviewer.

Part II – Major Issues: Key Experiments Required for Acceptance

Please use this section to detail the key new experiments or modifications of existing experiments that should be absolutely required to validate study conclusions. Generally, there should be no more than 3 such required experiments or major modifications for a "Major Revision" recommendation. If more than 3 experiments are necessary to validate the study conclusions, then you are encouraged to recommend "Reject".

Reviewer #1:

1. Lack of Supporting Data:

1) Organ-Parasite Load: The authors conclude about "organ-parasite" load in the 1st Results section without presenting any data on data. This omission makes the conclusion unsupported. Organ Weight: Similarly, the discussion on significant differences in organ weight is not backed by any data on organ weight, leading to unsupported claims.

Author Response: There are two issues raised by the reviewer that we would like to address.

Organ-Parasite Load: We want to clarify that the reviewer has somehow overlooked the data presented on organ parasite load utilizing two different experimental approaches. **In the Figure 1C.i. and 1C. iii., of the manuscript we have already provided the data related to organ parasite load. Figure 1C.i.** measures the expression of amastin in the spleen of infected mice and this is a well-accepted method reported by multiple groups to quantify organ parasite load (de Paiva, Grazielle-Silva 2015, PLoS pathogens ; Roy, Sarkar 2022, Parasitology). Apart from this, the organ-parasite load is also determined by Leishman-Donovan units (LDU), which is calculated as amastigote per nucleated cell * organ weight in mg (Khanra, Sarraf 2017, Scientific reports ; Mukherjee, Mukherjee 2012, PLoS neglected tropical diseases). We have also provided LDU values for all the experimental groups in **Figure 1C.iii**. Somehow the reviewer might have overlooked and missed both of these data.

Figure 1

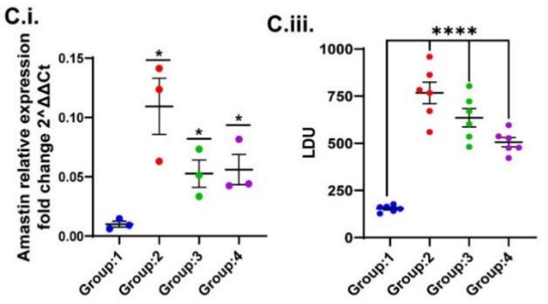

Figure 1: LD-R outcompetes LD-S in *in-vivo* competition infection. (C.i.) Relative amastin expression fold change (2^ΔΔCt) in the infected-spleen isolated 28 days pi from Groups:1-4 (N=3 for each group) was determined from qRT-PCR depicting the amastigote load in each set with respect to murine β-actin. Group:2, 3, and 4 showed significantly higher amastin relative expression as compared to Group:1 (*, P ≤ 0.05). No significant difference was observed between Group:2, Group:3, and Group:4 (ns, P > 0.05). Data presented as Mean ± SEM. **(C.iii.)** Graph showing LDU in each experimental set (Groups:1-4). N=3 for each group in duplicates for individual experimental

sets. Group:2, 3, and 4 showed significantly higher LDU as compared to Group: 1 (****, P ≤ 0.0001). Group:2 vs Group:3 (ns, P=0.1118), Group:2 vs Group:4 (**, P=0.0019), and Group:3 vs Group:4 (*, P=0.0395). Data presented as Mean ± SEM.

A. Organ Weight: Regarding the reviewer's comment on "organ weight", it has been clearly mentioned in lines 552 of the MS that spleen weight was measured and used to calculate the LDU (LDU= amastigote per nucleated cell * organ weight in mg) for each experimental group. Since we have provided the LDU, we didn't provide spleen weight separately, as it would have not added anything substantially new. However, as the reviewers have suggested we are providing the organ weight of individual spleen isolated from mice (**Figure R1**). We would also like the reviewer to note we have also provided visual representative images of the individual spleens from each experimental group in **Figure 1B.ii** reflecting variation in their sizes in the original MS, which should also substantiate their difference in weight. With all these data, we believe that we have convincingly pointed out that we haven't omitted any critical results that make our claim unsupported in any possible logical way.

Figure R1

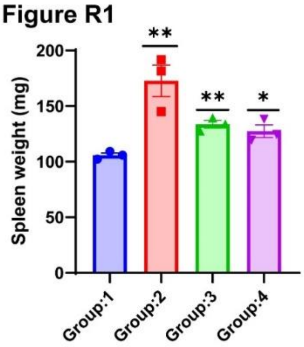

Figure R1: Bar graph representing infected mice spleen weight of different experimental sets (Group:1-4) expressed in mg (N=3). Group:1 representing LD-S-GFP, Group:2 representing LD-R, Group:3 representing LD-S-GFP: LD-R in 50:50 ratio, Group:4 representing LD-S-GFP: LD-R in 80:20 ratio. Group:2 (**, P ≤ 0.01), Group:3 (**, P ≤ 0.01), and Group:4 (*, P ≤ 0.05) show significant increase in spleen weight as compared to Group:1.

Figure 1

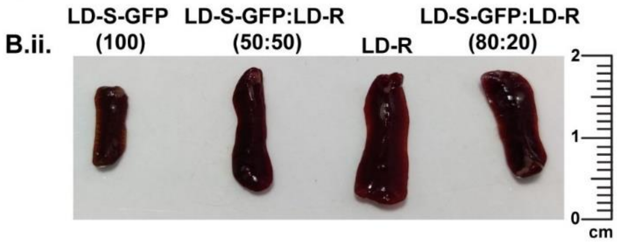

Figure 1: (B.ii.) Representative spleen image of infected-spleen 28 days pi from mice representing Groups: 1, 3, 2, and 4 (from left to right) with a scale bar (cm) at the right for differential comparison.

2) Inconsistent Methodology:

In Vitro vs. In Vivo Comparisons: For in vitro infection, a GFP susceptible strain and a RFP resistant strain are compared, but for in vivo experiments, a GFP susceptible strain is compared with a non-fluorescent resistant strain. The methodology should be consistent, and the same tagged resistant strain should be used in both in vitro and in vivo experiments to track both strains in microscopy and cytometry experiments.

Response: We understand and acknowledge this comment raised by the reviewer. However, we would like to clarify that it was an informed choice on our part not to use RFP-resistant strain in *in-vivo* competition experiments for a logical reason as follows:
We argue that since resistant strains show aggressive proliferation *in-vitro* (**Figure 1A. iv.**), we assume that the RFP-positive resistant parasite strain will possibly outcompete the GFP-positive susceptible population in infected mice, thus making it difficult to track GFP-positive population (even if it exists in a low level) from within a larger RFP-resistant population in competition experimental sets (Group:3 and Group:4). Hence, we chose an untagged resistant strain to make sure we could better track the GFP signal, even if it is severely diminishment in the *in-vivo* competition assay.
However, we acknowledge the concern raised by the reviewer and we agree with the reviewer that we need to repeat the experiment with RFP-tagged resistant strain as done in an *in-vitro* setup to further substantiate our claim. We request a chance to perform this experiment and request 1 month time to provide this data. We hope that the reviewer will understand our logic behind selecting untagged

resistant strains and we further infer that this does not amount to any gross conclusion of inconsistent methodology rather suggest an additional experiment which we can include in the revision.

3) Missing Data and Results:
No results are presented for the description on lines 156-158.
The data does not show differences among Groups 2, 3, and 4, contrary to the discussion on lines 143-148.
Essential data on parasite proliferation after 48 and 72 hours of in vitro infection and in vivo organ-parasite load, are missing.

Response: There are 3 points raised by the reviewer here, we will answer them one by one.

A. No results are presented for the description on lines 156-158.

Response: Lines 156-158 in the manuscript reads as, "Notably, it took approximately 11-13 days for Group:1, 5-7 days for Group:2, and 7-10 days for Group:3 and Group:4, for respective LD-promastigotes to emerge from the infected macerated spleens."
We didn't feel that it was necessary to plot the number of days in the graph as this has been already clearly mentioned in the results. But since the reviewer has suggested we plot the days it took for amastigote to promastigote differentiation for respective experimental sets (**Figure R2**). We will include this data in the supplementary file of the revised MS.

Figure R2

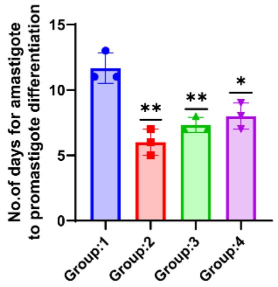

Figure R2: Bar graph showing the number of days it took for splenic amastigotes to differentiate into promastigotes in the culture for each experimental group (N=3). Group:2 and Group:3 showed significantly less number of days (**, $P \leq 0.01$) followed by Group:4 (*, $P \leq 0.05$) for amastigote-promastigote differentiation as compared to Group:1.

B. The data does not show differences among Groups 2, 3, and 4, contrary to the discussion on lines 143-148.

Response: Lines 143-148, reads as "This observation was in concurrence with amastin expression showing the highest splenic amastigote load for Group:2-infected-mice, followed by Group:3 and Group:4, all of which are significantly higher compared to Group:1-infected-mice **(Figure 1C.i.)**. Fluorescence microscopy of the splenic sections **(Figure 1C.ii.)** revealed GFP-positive amastigotes around the splenic MΦs in Group:1 whereas in Group:2, there was no signal of GFP as anticipated."
We would like to clarify that we in no way are trying to claim that there are significant differences in amastin expression among Group:2, Group:3, and Group:4 infected mice. We have clearly stated in **Figure 1C.i.,** amastigote load is highest for Group:2 infected mice, followed by Group:3 and Group:4 infected mice. And all these groups show significantly higher amastigote load compared to Group:1 infected mice (*, $P \leq 0.05$). Similarly, for **Figure 1C.iii.**, we observed that LDU was highest for Group:2 followed by Group:3 and Group:4, all of which are significantly higher than Group:1(****, $P \leq 0.0001$). Furthermore, the fluorescence microscopy images of **Figure 1C. ii** also clearly presents this difference with the highest reduction of GFP positive signal in Group:2-infected mice.
We apologize for the confusion and would modify this sentence as "This observation was in concurrence with amastin expression showing the highest splenic amastigote load for Group:2-infected-mice, followed by Group:3 and Group:4, all of which are significantly higher compared to Group:1-infected-mice **(Figure 1C.i.), with Group:2, Group:3 and Group:4 reflecting no significant difference between them**. Fluorescence microscopy of the splenic sections **(Figure 1C.ii.)** revealed GFP-positive amastigotes around the splenic MΦs in Group:1 whereas in Group:2, there was no signal of GFP as anticipated. Both in Group:3 and Group:4 infected-spleen there was a significant reduction in the number of GFP-positive amastigotes as expressed quantitatively in LDU, **with Group:3 showing slightly increased LDU than Group:4 (Figure 1C.iii.).**" We would also mention this clearly in the figure legend.

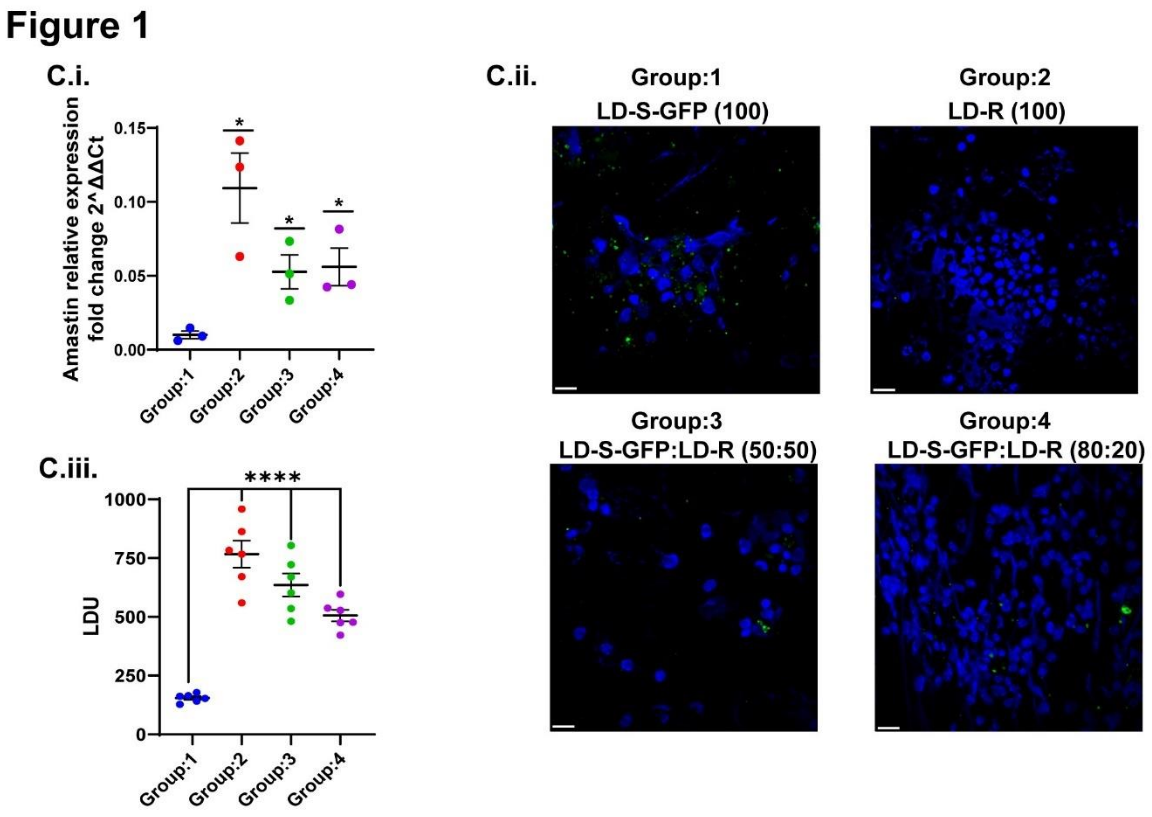

Figure 1: LD-R outcompetes LD-S in *in-vivo* competition infection. (C.i.) Relative amastin expression fold change (2^ΔΔCt) in the infected-spleen isolated 28 days pi from Groups:1-4 (N=3 for each group) was determined from qRT-PCR depicting the amastigote load in each set with respect to murine β-actin. Group:2, 3, and 4 showed significantly higher amastin relative expression as compared to Group:1 (*, $P \leq 0.05$). Data presented as Mean ± SEM. **No significant difference was observed between Group:2, Group:3, and Group:4 (ns, P > 0.05) (C.ii.)** Confocal images of the macerated whole spleen sample 28 days pi for experimental groups: 1-4 representing the GFP-amastigote load (small green dots) in each splenic sample. Scale bars indicate 20µm. **(C.iii.)** Graph showing LDU in each experimental set (Groups:1-4). N=3 for each group in duplicates for individual experimental sets. Group:2, 3, and 4 showed significantly higher LDU than group 1 (****, $P \leq 0.0001$). **Group:2 vs Group:3 (ns, P=0.1118), Group:2 vs Group:4 (**, P=0.0019), and Group:3 vs Group:4 (*, P=0.0395).** Data presented as Mean ± SEM.

C. Essential data on parasite proliferation after 48 and 72 hours of in vitro infection and in vivo organ-parasite load, are missing.

Response: In this entire study, we have clearly defined early time points as 4hrs post-infection and late time points as 24hrs post-infection for all the in-vitro experimental designs. We would like to clarify that the selection of these time points is deliberate, not random. We focused on these two time points because the trend in amastigote proliferation differences observed at 24 hours persists at 48 and 72 hours. Including additional data points would not provide new insights, so they were not included. However, to convince the reviewer we are providing this data (**Figure R3**) and this can be included in the supplementary figure.
Regarding the *in-vivo* organ-parasite load, in the MS lines 544-545 of the materials and methods section, it is clearly mentioned, “mice were sacrificed at 28days pi (for splenic amastigote load) or 24weeks pi (for establishing anemia model) as per experimental requirements.” It is very well-established that splenic parasite burden is started appearing around 14-28days pi (Cotterell, Engwerda 2000, Infection and immunity ; Lang, Ave 2000, Cellular microbiology). Hence, it is not possible to provide the data on 48-72hrs infection to determine *in-vivo* organ parasite load as suggested by the reviewer. We firmly believe that the reviewer will now be convinced that there is no 'missing data' described in the results section without supporting evidence.

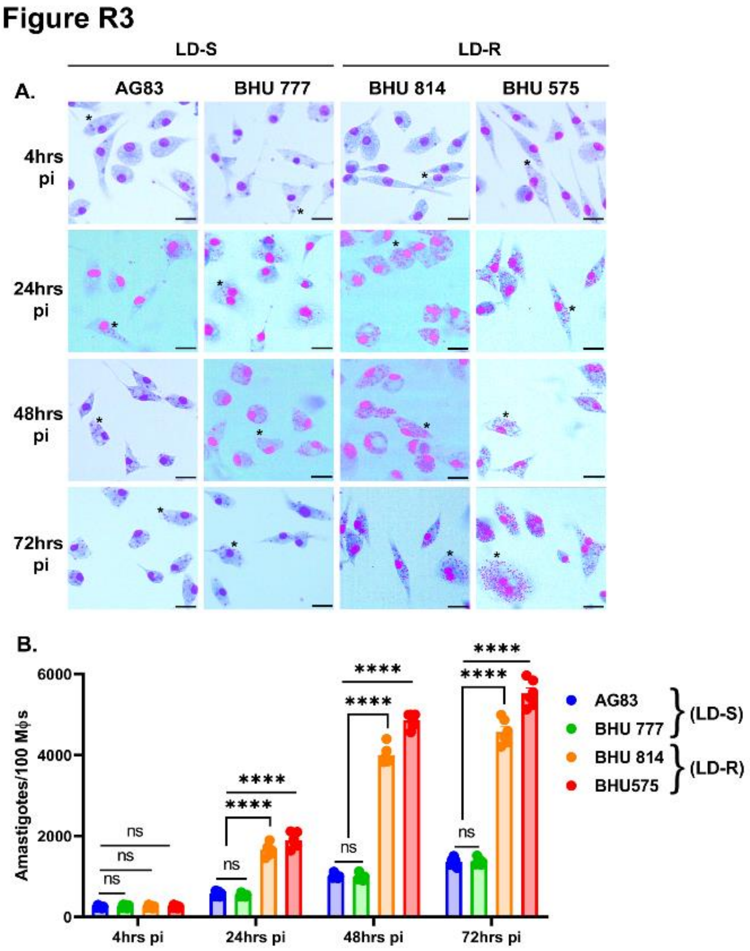

Figure R3: Antimony-resistant phenotype coincides with higher parasite burden if metacyclogenesis is kept as a constant factor. (A.) Giemsa-stained images of an equal number of AG83, BHU777 (representative LD-S) and BHU814, and BHU575 (representative LD-R) metacyclic promastigote infected-MΦs at 4hrs,24hrs, 48hrs, and 72hrs pi. One representative small nucleus of LD has been marked in (*) to show the infected-MΦs. **(B.)** Amastigotes/100 MΦs at 4hrs, 24hrs, 48hrs, and 72hrs pi were calculated taking different fields, and the data represents the Mean ± SEM of three independent experiments. **** denotes $P \leq 0.0001$ and ns denotes $P > 0.05$.

4) Poor Image Quality and Inadequate Controls:
Confocal images in Fig 1C.ii and FigS1D.i are of poor resolution and do not support any conclusions.
Figure 2A lacks scale bars and controls, making it difficult to interpret the results accurately.

Response: We would clarify each concern in a point-by-point manner.

A. Confocal images in Fig 1C. ii and FigS1D.i are of poor resolution and do not support any conclusions.

Response: We would like to point out that there are multiple confocal images which has been provided in this MS **(Figure 2A.i., 2B.i., 3D.ii.a., 4A.i., 4Bi., 4C.i.a., 4D.i., 4D.ii., 4E.i.a., 5A.i., 5A.iii., 5B.i.a., 6B.ii., 6C, S3A.i., S3B, S3C, S3D, S4A.ii.,** and **SB. ii. a)** out of which reviewer have pointed out only two images as poor-quality images**, and reason behind this is entirely technical as we have explained in the next section**.
Both **Figure 1C. ii** and **S1D.i** are representative fluorescence images of the whole spleen macerate of the infected murine spleen in lower magnification (20X). This was performed specifically to capture the GFP-positive amastigote signal in a wide region of view. We have clearly mentioned these in the Figure legends of the respective figures, "Confocal images of the macerated spleen sample 28 days pi for

experimental groups: 1-4 representing the GFP-amastigote load (small green dots) in each splenic sample. Scale bars indicate 20µm.**”.** Since these two images represent the whole splenic macerates with low magnification, it is understandable that these two particular images are of not as superior quality as compared to all the other images provided in the MS. However, we believe these images still clearly represent the differential GFP expression among different experimental groups and convey the required message effectively. We hereby have included the enlarged images for better representation. Also, we would like to point out that the conclusion of this image is also supported independently by multiple other results (**Figure 1B.ii.**, **1C.i.**, **1C.iii** and **1C. iv.)** all of which have indicated towards same conclusion. If the reviewer still feels these two images are not good enough, we can remove or replace them.

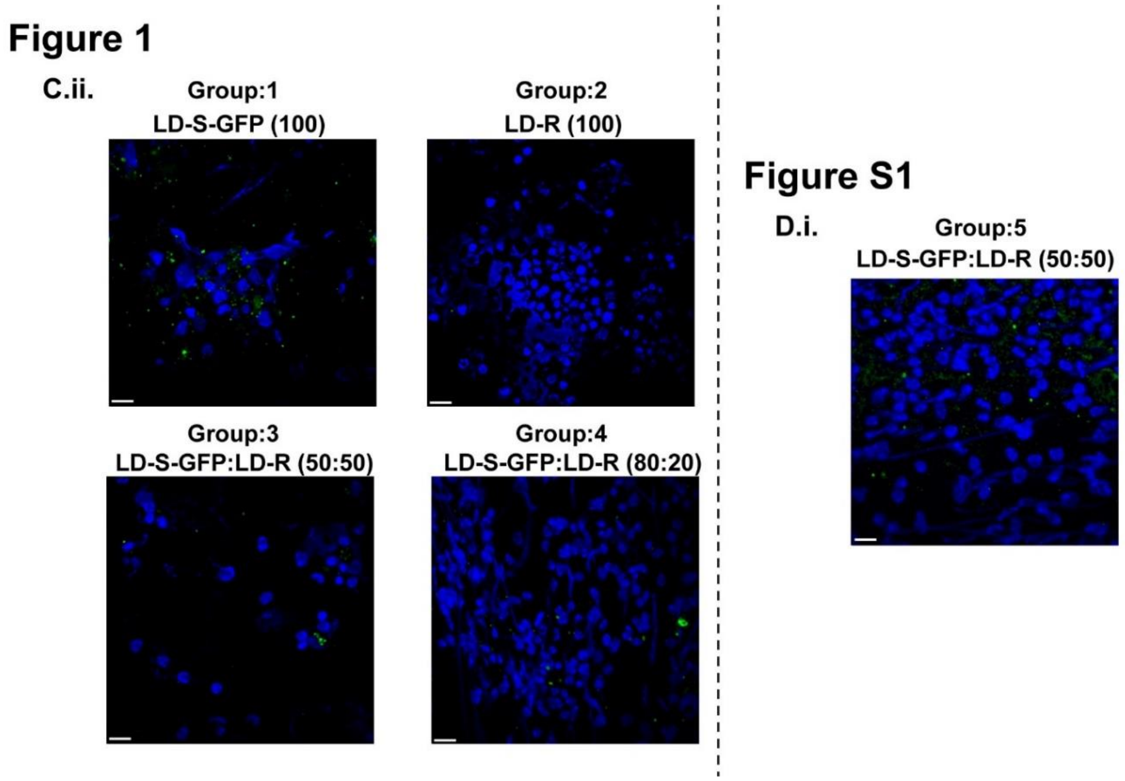

Figure 1C.ii. and Figure S1D.i.: Confocal images of the macerated whole spleen sample 28 days pi for experimental groups: 1-4 and 5 respectively representing the GFP-amastigote load (small green dots) in each splenic sample. Scale bars indicate 20µm.

B. Figure 2A lacks scale bars and controls, making it difficult to interpret the results accurately.

Response: Figure 2A represents two figures (**Figure 2A.i. and 2A.ii.**). We believe the comment regarding the scale bar is related to **Figure 2A.i.** We would like to thank the reviewer for pointing this out, actually if observed closely, **Figure 2A.i.** has scale bars (in black) and has been mentioned in the figure legend (please see the zoomed section). But we agree this is probably not visible to the reviewer as well as readers and we apologize for not making it more prominent. Hence, we are adding it manually outside the image for better visibility (**Figure R4A**).

Figure 2

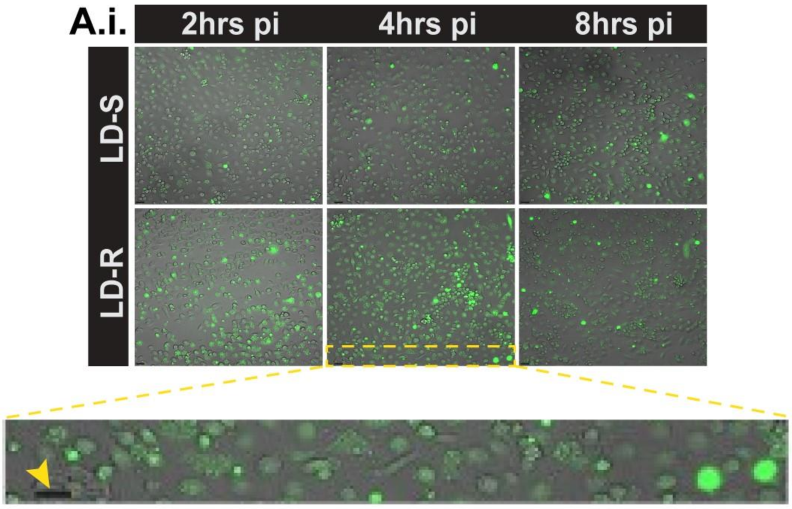

Figure 2: (Ai.) 20X-magnification live-cell confocal images of H_2DCFDA-stained (green) LD-S and LD-R infected-MΦs at early hours (2, 4, and 8hrs pi) with zoomed area (demarcated in yellow dotted area) showing scale bar of 20µm (marked in yellow arrow).

Figure R4

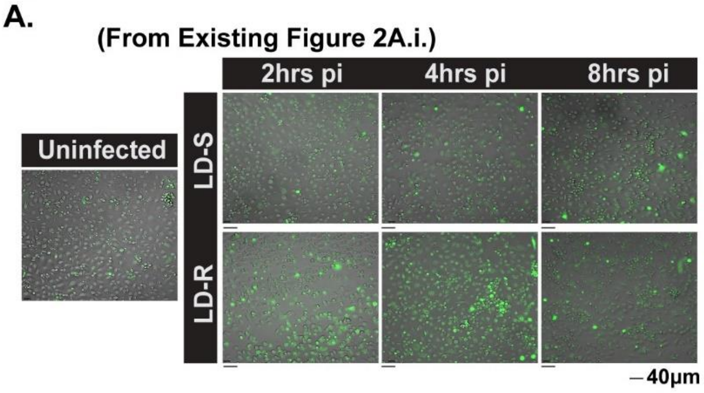

Figure 2: Monitoring of ROS level by Confocal live-cell imaging. (Ai.) 20X-magnification live-cell confocal images of H_2DCFDA-stained (green) LD-S and LD-R infected-MΦs at early hours (2, 4, and 8hrs pi). Black scale bar outside the image represents 40µm.

Regarding the 'controls' mentioned by the reviewer, we would like to point out that as this Figure 2A specifically compares an inherent metabolic shift between LD-R and LD-S at multiple time points, we believe it was not an absolute necessary to include additional uninfected control in this figure to reduce the figure burden, although we had that experimental sets all along. Similarly, for flow cytometry estimation of the DCF-FITC-A positive population (**Figure 2A.ii.**), we had uninfected unstained peritoneal macrophage control to gate the macrophage population and 100µM H_2O_2-treated macrophage positive control (Srivastav, Basu Ball 2014, The Journal of biological chemistry) for gating the DCF-FITC-A positive population. However, we opted to include only LD-S and LD-R comparison to reduce the figure burden. As suggested by the reviewer, we have now included these additional experimental sets in the revised **Figure R4A** and **R4B,** and will include them in the revised manuscript.

Figure R4: Monitoring of ROS level by Flow cytometer-based approach. (B.) Representative Flow cytometry images of half offset histogram plot representing live-cell quantification of DCF fluorescence in FITC-A filter in LD-S and LD-R infected MΦs at 2hrs, 4hrs, and 8hrs pi. Uninfected unstained control was used for gating the macrophage population and 100µM H_2O_2 treated macrophage positive control (1hr treatment) was used to gate the DCF-positive population in the FITC-A channel. The grey area demarcates the DCF-FITC-A-positive population. H_2O_2-treated macrophages showed significant ROS outbursts as compared to uninfected unstained control (****, $P \leq 0.0001$). LD-R 2hrs pi showed no significant change as compared to LD-S 2hrs pi (ns, $P > 0.05$), while at 4hrs pi, LD-R showed significant ROS outburst compared to LD-S infection (*, $P \leq 0.05$), and at 8hrs pi LD-R shows a significant drop in ROS level as compared to LD-S infection (***, $P \leq 0.001$), N=3 independent biological repeats.

Figure R4

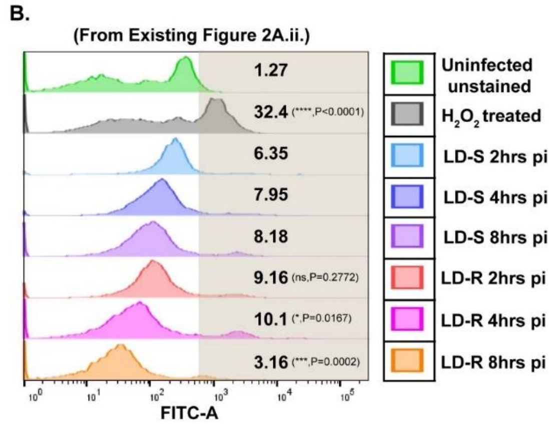

5) Unsupported Conclusions:

ROS Levels: Conclusions on ROS levels differences between LD-S and LD-R strains are not supported by the presented data and statistical analysis (lines 168-171). Ferritin Levels: Conclusions on Ferritin levels are not supported by the densitometry analysis of the western blot in Fig. 3B. Quantification of HO-1 levels and biological replicates for the western blot analysis are also missing. Luciferase Activity: The conclusion that luciferase activity modulation is exclusive to LD-R is not accurate, as activity modulation is also observed in LD-S.

Response: Again there are 3 concerns and we have addressed individual concerns one by one.

A. ROS Levels: Conclusions on ROS levels differences between LD-S and LD-R strains are not supported by the presented data and statistical analysis (lines 168-171).

Response: Line 168-171 reads as, "However, LD-R-infected-MΦs exhibited higher ROS levels at 2hrs and 4hrs pi compared to LD-S-infected-MΦs, which flattened out by 8hrs pi **(Figure 2A.i. and A.ii., S1E)**. However, incubation of MΦs with killed-LD-R failed to generate substantial ROS, indicating that replicating LD-R is required for ROS generation **(Figure S1E)**."

We have presented the Flow data as a histogram plot and not as a graph for better representation but we agree we should have included the p value. We have rectified this mistake and have already included the same in **Figure R4B** and in the Figure legends which clearly support the conclusion on ROS level.

We would also like to bring to the reviewer's attention, in addition to Confocal and Flow cytometer-based quantifications, we have also performed Fluorescence plate reader-based quantification of ROS, which is presented in **Figure S1E**, the statistical significance of both these results are provided which again clearly supports the conclusion of the sentence (168-171) referred by the reviewer. Apart from this we have also included statistical analysis also for the ROS measurement represented in Figure S1F and S1G (not requested by the reviewer) to further strengthen our point. With these evidences from multiple independent experiments, we strongly feel that we will be able to convince the reviewer.

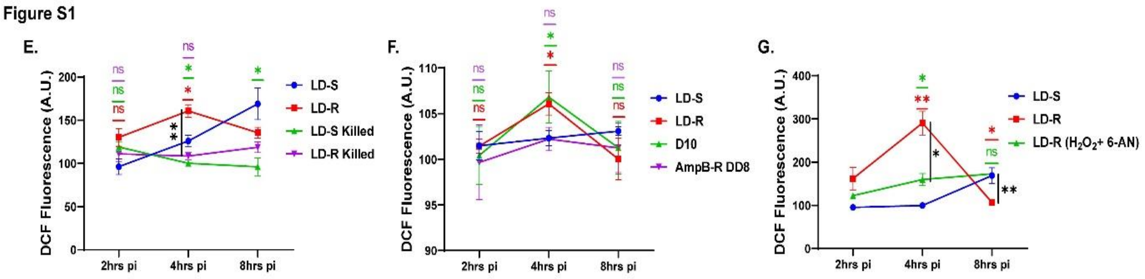

Figure S1. ROS level monitoring (E.) DCF-fluorescence quantification of LD-S, LD-R, heat-killed-LD-S, and heat-killed-LD-R infected-MΦs at early hours. LD-R killed experimental sets at 4hrs pi significantly fail to generate ROS as compared to LD-R infected-MΦs at 4hrs pi (**, P ≤ 0.01). **(F.)** DCF-fluorescence quantification of MΦs infected with LD-S or LD-R or D10 or AmpB-R DD8 **(G.)** DCF-fluorescence quantification of MΦs infected with LD-S or LD-R, or $NADPH^{exh}$-LD-R by H_2O_2+6-AN treatment. LD-R (H_2O_2+6-AN) significantly failed to generate ROS as compared to LD-R infected-MΦs at 4hrs pi (*, P ≤ 0.05). At 8hrs, LD-R infected- MΦs shows a significant drop in ROS level compared to LD-R (H_2O_2+6-AN) (**, P ≤ 0.01), which remains unchanged like 4hrs pi. P-value summary is color-coded corresponding to the color of individual experimental sets and all the analysis is performed compared to LD-S infected MΦs at individual time points except for the one in black in Figure S1E and S1G. ** denotes P ≤ 0.01, * denotes P ≤ 0.05, and ns denotes P > 0.05

B. Ferritin levels: Conclusions on Ferritin levels are not supported by the densitometry analysis of the western blot in Fig. 3B. Quantification of HO-1 levels and biological replicates for the western blot analysis are also missing.

Response: We are providing Densitometry analysis and the biological replicates as requested by the reviewer (**Figure R5**). We would like to mention that providing densitometry analysis for all the western blots, particularly for those where the difference in band intensity is clearly visible by just looking at them will result in a lot of additional graphs making the figure dense. Hence if the reviewer feels that this is absolutely necessary we would like to request to allow us to include all the densitometry analysis of the western blots in the supplementary figure rather than putting them as the main figure.

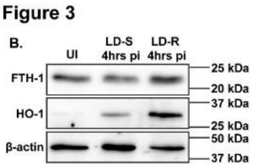

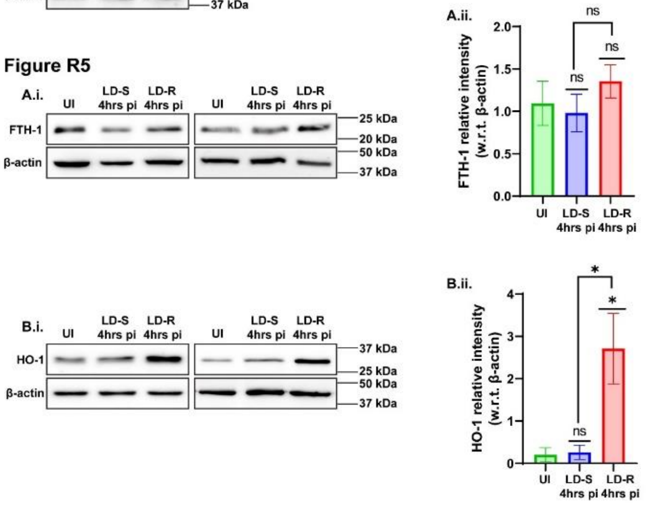

Figure R5: Western blots of Ferritin (FTH-1) and Heme oxygenase 1 (HO-1) in triplicates (**Figure 3B**, **R5A.i.** and **R5B.i.**) and the representative bar graphs of relative intensity fold change with respect to β-

actin (Figure R5A.ii. and R5B.ii. respectively). **(R5A.ii.)** No significant change in FTH-1 was observed in any experimental conditions (ns, $P > 0.05$). **(R5B.ii.)** Significant upregulation of relative intensity of HO-1 is observed in LD-R 4hrs pi as compared to both UI and LD-R 24hrs pi (*, $P \leq 0.05$) whereas no significant change is observed in LD-S 4hrs pi as compared to UI (ns, $P > 0.05$).

C. Luciferase Activity: The conclusion that luciferase activity modulation is exclusive to LD-R is not accurate, as activity modulation is also observed in LD-S.

Response: We completely agree with the reviewer on this. Some level of change is although minor is also noticed for LD-S infection (**Figure 3E**). We regret that we have missed this and thank the reviewer for pointing this out. We have addressed this by modifying the conclusion as suggested by the reviewer and will include this in the result and discussion section of the revised MS. "Finally, Luciferase assay with wild-type, or truncated promoter constructs: Site $A^{-/-}$ (-4/-635) and Site $B^{-/-}$ (-636/-1377) in the presence of LD-R and LD-S infection, further confirmed specific interaction of p50/c-Rel with Site A of HO-1 promoter resulting in its significant upregulating in LD-R-infected MΦs **(Figure 3E). Interestingly we did observe some luciferase activity modulation in HO-1 promoter construct in response to LD-S-infection, probably owing to the effect of some other transcription factors having binding sites within HO-1 promoter as we observed a slightly upregulated HO-1 even in LD-S-infected MΦs as compared to uninfected control** (**Figure 3B**).

6) Inadequate Statistical Analysis:

Statistical analyses for several conclusions, including anemia-related findings, are not presented. Specifically, the HCT differences between LD-S and LD-R are not statistically significant (t test done by myself).

Response: We have clearly mentioned the statistical analysis including the number of biological repeats and *p*-value for all our results in respective figure legends. Regarding anemia-related findings, we have added the statistical analysis in **Figure 7A.iii**. Additionally, we would also add a paragraph in the materials and method section to avoid any further doubts on statistical analysis as follows:

"**Statistical Analysis**
Each experiment was performed in triplicates, independently. The results are expressed as mean ± SEM (in some cases as ± SD as specifically mentioned in the figure legends). Statistical significance was calculated by performing an unpaired t-test in GraphPad Prism 8.0.2, and a P-value <0.05 was considered statistically significant. Values were considered most significant (represented as: ****) if $P \leq 0.0001$, extremely significant (represented as: ***) if $P \leq 0.001$, very significant (represented as: **) if $P \leq 0.01$, or significant (represented as *) if $P \leq 0.05$; and non-significant (represented as: ns) if $P > 0.05$."

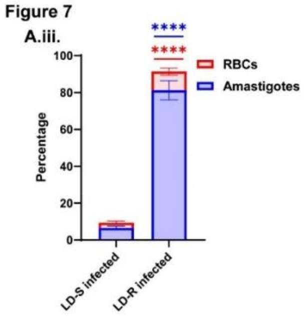

Figure 7: (A.iii.) Stacked bar plots showing % of amastigotes and RBC engulfed by erythrophagocytes in LD-S and LD-R infected-spleen. LD-R infected spleen shows significantly higher RBCs engulfed and amastigote burden as compared to LD-S infected spleen at 24weeks pi (****, $P \leq 0.0001$).

Regarding the specific concern raised by the reviewer for the significance of the HCT difference between LD-S and LD-R infected mice, we would like to mention that it has been calculated using GraphPad Prism 8 software. GraphPad Prism is a well-accepted software for performing statistical analysis and is almost used by all researchers in the field. The P value comparing HCT between LD-S and LD-R was 0.0487, which is significant (please see the attached screenshot). We are not sure how without raw data points, the reviewer was able to calculate the statistical significance but we are highly confident about our statistical analysis for all our data and will be willing to share all these files if requested.

Unpaired t test	
Table Analyzed	HCT
Column C	LD-R infection
vs.	vs.
Column B	LD-S infection
**Unpaired t test**	
P value	0.0487
P value summary	*
Significantly different (P < 0.05)?	Yes
One- or two-tailed P value?	Two-tailed
t, df	t=2.803, df=4
**How big is the difference?**	
Mean of column B	35.37
Mean of column C	31.67

Figure: Screenshot showing unpaired t-test analysis performed in GraphPad Prism for HCT in LD-R infected blood vs LD-S infected blood at 24weeks pi.

7) Methodological Concerns:

The percentages of metacyclics shown in Fig 1Ai, Aii and Fig S1A are before or after sorting? Most published works report percentages below 40% before purification.
Calcein is not a reliable method for LIP quantification in this context (doi: 10.1042/BJ20061840, among others).
Many western blot-related conclusions are quantitative, yet the manuscript does not report how these quantifications were performed or the number of replicates.

Response: We are providing clarification for each of these points which should essentially clear out any doubts of the reviewer regarding all the methodological issues and will be able to convince him about stringency of all the methods used in this study.

A. The percentages of metacyclics shown in Fig 1Ai, Aii and Fig S1A are before or after sorting? Most published works report percentages below 40% before purification.

Response: We have no conflict against this comment of the reviewer. In fact, we also observed a higher percentage of metacyclics in resistant strains as compared to sensitive ones as previously reported by other groups (Ouakad, Vanaerschot 2011, Parasitology). That is why as mentioned in the materials and method section, lines 728-730, "Metacyclic promastigotes were sorted using Beckman Coulter Cytoflex srt by gating *FSC-A* X *SSC-A* where FSC^{low} represents the metacyclic population and FSC^{high} represents the procyclic population". Next, a portion of these sorted metacyclic promastigotes was further checked on the Flow cytometer capturing 20,000 events which is represented in **Figure 1A.i.**, **1A.ii.**, and **S1A** signifying uniform metacyclics between LD-S and LD-R for performing downstream experimental studies. We do agree with the reviewer that for the clarity of readers, this information also needs to be included in Figure legends of the respective figure.

"**Figure 1: Representative flow cytometer images (BD LSRFortessaTM Cell Analyzer) showing the % of** metacyclics and procyclic LD-promastigote population in the culture of **(A.i.)** GFP-expressing antimony-sensitive (LD-S-GFP) and **(A.ii.)** RFP-expressing antimony-resistant (LD-R-RFP) **after uniform sorting** in Beckman Coulter Cytoflex srt. FSC^{low} (left) represents the metacyclic population and FSC^{high} (right) represents the procyclic population."

"**Figure S1.A. Representative flow cytometer images (BD LSRFortessaTM Cell Analyzer) showing the** % of metacyclic promastigote in AG83, BHU777 (7th-day culture); BHU814, BHU575 (5th-day culture) **after uniform sorting** in Beckman Coulter Cytoflex srt. **FSC^{low} (left) represents the metacyclic population and FSC^{high} (right) represents the procyclic population**"

B. Calcein is not a reliable method for LIP quantification in this context (doi: 10.1042/BJ20061840, among others).

Response: This is an important issue and we would like to thank the reviewer for raising this. Firstly, we would like to mention that multiple reports have used Calcein-based assays for the labile iron pool (LIP) monitoring (Prus and Fibach 2008, Cytometry A ; Riedelberger, Penninger 2020, Cell host & microbe). However, we do appreciate the point the reviewer is making here. Please note we have also cited the article that the reviewer has referred to(Tenopoulou, Kurz 2007, The Biochemical journal) in his comment. We would like to bring to the reviewer's attention that this article states that "Calcein fluorescence is quenched following chelation of low-mass labile iron, and the degree of quenching gives an estimate of the amounts of chelatable iron." Furthermore, this paper also points out that, "calcein does not readily chelate iron at lysosomal pH (pH 4–5), the calcein-AM method will not demonstrate all intracellular low-mass iron, but only a fraction of it." Since it is well reported that Lipophosphoglycan of *Leishmania donovani* inhibits phagolysosomal maturation at an early stage of infection to prevent acidification (Vinet, Fukuda 2009, PLoS pathogens ; Verma, Rastogi 2017, PLoS pathogens), we argue that this allows a reliable quantification of LIP by Calcein during early stage of Leishmania infection (at 4hrs pi represented in **Figure 4A.i.**, **A.ii.**, **B.i.**, **D.ii.**, and **S3C**).
We would also like to bring to the reviewer's attention that for this particular reason, we have not used calcein-based quantification at the late hours of infection (24hrs pi) when the parasitophorous vacuole becomes acidic due to lysosomal fusion. For late hours thus we have quantified LIP by using another well-accepted method i.e. Ferrozine-based colorimetric assay (Riemer, Hoepken 2004, Anal Biochem ; Banerjee and Datta 2020, Cellular microbiology ; Banerjee and Datta 2023, The Journal of biological chemistry) . It is to be further noted that both these independent quantification methods yielded similar observations at both 4hrs and 24hrs pi (**Figure 4 B.ii. and Figure S2 A**) which further validate this methodology and strengthens our claim. Since these multiple quantifications provided similar results, this should essentially remove any doubts regarding the methodology.

C. Many western blot-related conclusions are quantitative, yet the manuscript does not report how these quantifications were performed or the number of replicates.

Response: As we have already mentioned earlier in response to concern number 5, we would again like to clarify that we have not included the densitometry analysis for the western blot as the change in band intensities was prominent enough to point out the quantitative differences. However, as all the images were already available with us it was really not an issue to include the densitometry analysis for all the western blots used in this study (**Figure R6 and RS1**). This should be sufficient to remove any doubts associated with the western blot expression level.

Figure R6

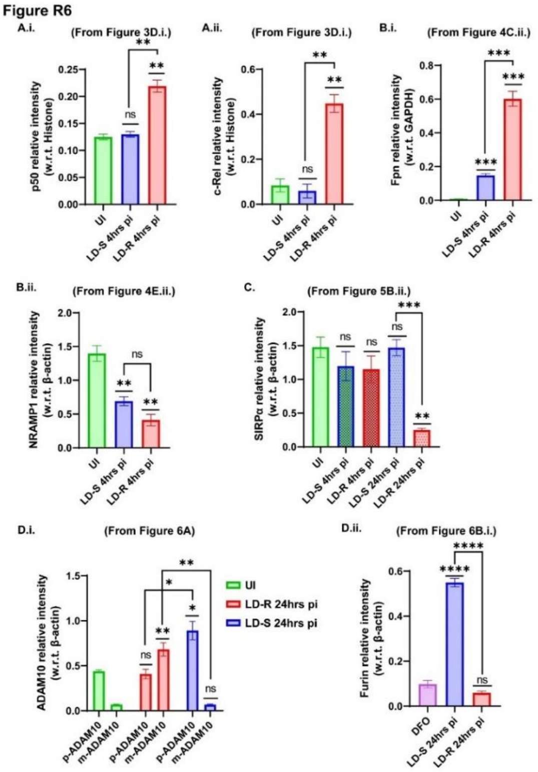

Figure R6: Western blot analysis showing the relative intensity of proteins i.e. fold change with respect to housekeeping control *i.e.* Histone for nuclear fraction, GAPDH and β-actin for whole cell lysate. **(A.i. and A.ii.)** Significant enrichment of nuclear p50 and c-Rel respectively observed in LD-R 4hrs pi as compared to UI and LD-S 4hrs pi ($P \leq 0.01$ marked as **) whereas LD-S 4hrs pi shows no significant change (ns) compared to UI ($P > 0.05$). **(B.i.)** Significant upregulation of Ferroportin (Fpn) in both LD-S and LD-R 4hrs pi (***, $P \leq 0.001$) as compared to UI, with significant upregulation in LD-R 4hrs pi as compared to LD-S 4hrs pi (***, $P \leq 0.001$). **(B.ii.)** Significant downregulation of relative intensity of NRAMP1 in both LD-S and LD-R at 4hrs pi as compared to UI, whereas no significant change is observed in between both these infected experimental conditions. **(C.)** Significant downregulation of relative intensity of SIRPα in LD-R 24hrs pi as compared to UI (**, $P \leq 0.01$) and LD-S 24hrs pi (***, $P \leq 0.001$). **(D.i.)** Significant upregulation of mature- ADAM10 (m-ADAM10) in LD-R 24hrs pi as compared to both UI and LD-S 24hrs pi (**, $P \leq 0.01$) whereas significant upregulation of precursor-ADAM10 (p-ADAM10) is observed in LD-S 24hrs pi as compared to both UI and LD-R 24hrs pi (*, $P \leq 0.05$). **(D.i.)** Significant enrichment of Furin observed in LD-S as compared to both DFO-treated control and LD-R 24hrs pi (****, $P \leq 0.0001$) whereas no change is observed between LD-R 24hrs pi and DFO-treated control (ns, $P > 0.05$). Each densitometry analysis is presented as a bar graph of Mean ± SEM for 3 biological replicates.

Figure RS1

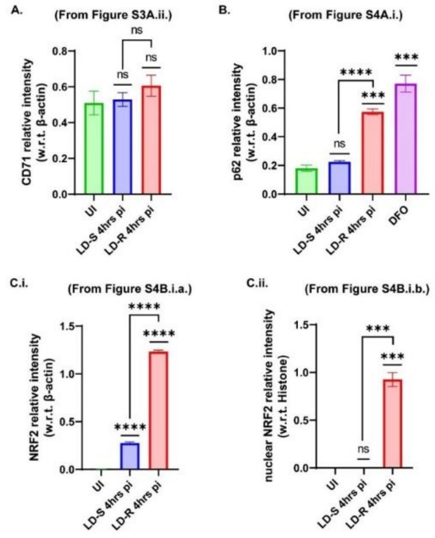

Figure RS1: Western blot analysis showing the relative intensity of proteins i.e. fold change with respect to housekeeping control *i.e.* Histone for nuclear fraction and β-actin for whole cell lysate. **(A.)** No significant change in the relative intensity of CD71 in UI, LD-S 4hrs pi, and LD-R 4hrs pi ($P > 0.05$). **(B.)** Significant upregulation of p62 in both LD-R 4hrs pi and DFO-treated control as compared to UI (***, $P \leq 0.001$). Also, significant upregulation in p62 expression in LD-R 4hrs pi as compared to LD-S 4hrs pi (****, $P \leq 0.0001$). **(C.i.)** Significant upregulation of NRF2 in both LD-S and LD-R 4hrs pi (****, $P \leq 0.0001$). Also, there is significant upregulation in LD-R 4hrs pi vs LD-S 4hrs pi (****, $P \leq 0.0001$). **(C.ii.)** Significant upregulation of nuclear-NRF2 in both LD-R 4hrs pi as compared to both UI and LD-S 4hrs pi (***, $P \leq 0.001$). Each densitometry analysis is represented as a bar graph of Mean ± SEM for 3 biological replicates.

<u>Reviewer #2:</u> The manuscript entitled "Oxidative stress-driven enhanced iron production and scavenging through Ferroportin reorientation worsens anemia in antimony-resistant Leishmania donovani Infection" has investigated possible mechanisms that aid the persistence of Antimony resistant L. donovani parasites, in the Indian subcontinent even after the cessation of antimony treatment in the clinics. These parasites persist better in the host than antimony-sensitive ones. They show that resistant parasites to antimonials scavenge iron more efficiently in the parasitophorous vacuole, than sensitive ones and hence can replicate better as amastigotes. With the use of an antioxidant NAC, they show ROS are required for the survival of the resistant parasites and this was associated with upregulation of OH-1, but this increase was associated with a downmodulation of cytoplasmic Fe+2, contrary to what it was believed.

This was associated with an increase of iron, at 4 hr pi of LD-R-Parasitophorous Vacuole. The latter was associated with targeted redistribution of the MΦ membrane iron-exporter, Ferroportin, around LD-R-PV, where the authors say that this causes a downmodulation of cytoplasmic iron and increases the activation of p62/SQSTM1. This is known to degrade the inhibitor of Nrf1 Keap1, facilitating the translocation of Nrf1 to the nucleus, a transcription factor known to initiate the antioxidant response. This iron insufficiency results in increased erythrophagocytosis, via SIRPα degradation, mediated by furin and ADAM10. The authors suggest that erythrophagocytosis is the cause of anaemia, observed in patients infected with LD-R parasites, as it is the case for infected mice with LD-R parasites. This manuscript is novel and has a lot of different experimental procedures and explains contradicting observations in macrophage-Leishmania interactions, like the activation of ROS for the benefit of the parasite. However the presentation is long and complex and the authors need to reassure the readers about the quality of some experiments (described in major comments). Overall, the positive elements of this manuscript make it worth for publication, if the major comments are addressed.

Author Response: We sincerely thank the reviewer for the positive assessment of our work and appreciate its novelty and the different experimental approaches used to explain contradicting observations.

Reviewer #2: A lot of "unwanted information" which does not contribute to the final conclusion (ie increased iron pool in PV with lower amounts in cytoplasm which contributes to increase in erythrophagocytosis). I would present the result with the low iron HO-1 upregulation in the supplemental material. I would like to see a simplification and more results in the supplemental figures. For some experiments like the transfection of RAW macrophages with dual reporter luciferase, I would like the authors to comment as it is in general established the macrophages cannot be easily transfected with DNA. Also, as the authors present transcriptomics results provided as a service, but there is no clear mention of the quality of these data in the main MS (number of replicas etc). Also, many of these factors that are upregulated or downmodulated, apart from the Iron pathway affect the whole macrophage function (ie HO-1 causes a M2 phenotype in macrophages and hence allows the growth of intracellular parasites). In the discussion, it would be great to see some description between these results and the macrophage programs (inflammatory M1, anti-inflammatory M2).

Author Response:
The reviewer has raised a few concerns which we would like to address pointwise:

A. A lot of "unwanted information" which does not contribute to the final conclusion (ie increased iron pool in PV with lower amounts in cytoplasm which contributes to increase in erythrophagocytosis). I would present the result with the low iron HO-1 upregulation in the supplemental material.

Response: We agree with this assessment of the reviewer. We have added much information and additional experimental approaches to the main figures with the intention of validating every observation with additional approaches. However, we do feel that as a consequence, the figures have become much dense and may have resulted in reviewer 1 overlooking a few of the results which were already present in the MS. Hence, as per the reviewer's suggestion, we will update the main figures in the revised manuscript and transfer additional data to the supplementary section to make it as simple and crisp and reduce the data load.

B. For some experiments like the transfection of RAW macrophages with dual reporter luciferase, I would like the authors to comment as it is in general established the macrophages cannot be easily transfected with DNA.

Response: For the Dual-Luciferase assay, as correctly pointed out by the reviewer it's very difficult to achieve significant transfection in primary macrophages. Hence, we have used RAW 264.7

macrophage cell line which has been previously well-reported for achieving successful transfection using lipofectamine (Mukherjee, Mukhopadhyay 2013, Proceedings of the National Academy of Sciences of the United States of America ; Kang, Kwon 2014, Gene Ther) and we also observed the same result. We will add this as a note in the figure legend of the revised MS.

C. Also, as the authors present transcriptomics results provided as a service, but there is no clear mention of the quality of these data in the main MS (number of replicas etc).

Response: We agree and would like to elaborate on the transcriptomics results. We have attached the screenshot of the QC report indicating the quality of this data for your reference and the QC table as follows:

Library QC Report

**Kit used for Extraction**	NEBNext® Ultra™ II Directional RNA Library Prep Kit for Illumina
**Library QC Details**	Qubit 4.0 fluorometer(Thermofisher #Q33238)<br>Tapestation 2200 (Agilent)
**Sequencing Chemistry**	150XPE
**Sequencing Platform**	Nova Seq 6000
**Conclusion**	Pass
**Remarks**	Remarks

Library QC table

SI	Sample Name	Qubit ng/ul	QC Status	Volume (µL)
1	UI-1	11.2	PASS	15
2	UI-2	9.6	PASS	15
3	LD-S 4hrs pi-1	16.3	PASS	15
4	LD-S 4hrs pi-2	14.3	PASS	15
5	LD-R 4hrs pi-1	6.59	PASS	15
6	LD-R 4hrs pi-2	11.9	PASS	15
7	LD-S 24hrs pi-1	5.36	PASS	15
8	LD-S 24hrs pi-2	7.96	PASS	15
9	LD-R 24hrs pi-1	12.6	PASS	15
10	LD-R 24hrs pi-2	4.74	PASS	15

The number of replicates used in this study is 2. In brief, we have used the average normalized counts of the replicates using DESeq2 R packages for producing the heatmap. We would like to update the RNAseq materials and methods (lines 629-631) in the revised MS as follows: "Normalized counts (n=2) were obtained from the DESeq2 package and averaged normalized counts were used to develop heatmaps of a few selected genes using gplots and RColorBrewer."

D. Also, many of these factors that are upregulated or downmodulated, apart from the Iron pathway affect the whole macrophage function (ie HO-1 causes a M2 phenotype in macrophages and hence allows the growth of intracellular parasites). In the discussion, it would be great to see some description between these results and the macrophage programs (inflammatory M1, anti-inflammatory M2).

Response: We appreciate the reviewer's insightful suggestion and agree that it would be useful to look into factors apart from the iron pathway that are significantly upregulated or downregulated affecting the overall macrophage programming and eventually promoting the aggressive growth of intracellular-resistant parasites. As per the reviewer's suggestion, we would look into these pathways and add a brief paragraph in the discussion section of the revised manuscript.

Part III – Minor Issues: Editorial and Data Presentation Modifications

Please use this section for editorial suggestions as well as relatively minor modifications of existing data that would enhance clarity.

Reviewer #1: (No Response)

Reviewer #2: Some typos and grammatical errors. Ie line 407 intracellular proliferation

(requires a full stop). I would also like to see an abbreviation list. Also in the results section it is not always easy to understand if some experiments are performed in promastigotes, amastigotes or macrophages.

Author Response: We thank the reviewer for pointing out this typo in line 407 which we somehow overlooked. We will add the full stop in the revised manuscript. We would further look into the whole manuscript to fix any sort of typos and grammatical errors in the revised manuscript.

REFERENCES:

1. de Paiva RM, Grazielle-Silva V, Cardoso MS, Nakagaki BN, Mendonca-Neto RP, Canavaci AM, et al. Amastin Knockdown in Leishmania braziliensis Affects Parasite-Macrophage Interaction and Results in Impaired Viability of Intracellular Amastigotes. PLoS pathogens. 2015;11(12):e1005296.
2. Roy M, Sarkar D, Chatterjee M. Quantitative monitoring of experimental and human leishmaniasis employing amastigote-specific genes. Parasitology. 2022;149(8):1085-93.
3. Khanra S, Sarraf NR, Das AK, Roy S, Manna M. Miltefosine Resistant Field Isolate From Indian Kala-Azar Patient Shows Similar Phenotype in Experimental Infection. Scientific reports. 2017;7(1):10330.
4. Mukherjee S, Mukherjee B, Mukhopadhyay R, Naskar K, Sundar S, Dujardin JC, et al. Imipramine is an orally active drug against both antimony sensitive and resistant Leishmania donovani clinical isolates in experimental infection. PLoS neglected tropical diseases. 2012;6(12):e1987.
5. Cotterell SE, Engwerda CR, Kaye PM. Enhanced hematopoietic activity accompanies parasite expansion in the spleen and bone marrow of mice infected with Leishmania donovani. Infection and immunity. 2000;68(4):1840-8.
6. Lang T, Ave P, Huerre M, Milon G, Antoine JC. Macrophage subsets harbouring Leishmania donovani in spleens of infected BALB/c mice: localization and characterization. Cellular microbiology. 2000;2(5):415-30.
7. Srivastav S, Basu Ball W, Gupta P, Giri J, Ukil A, Das PK. Leishmania donovani prevents oxidative burst-mediated apoptosis of host macrophages through selective induction of suppressors of cytokine signaling (SOCS) proteins. The Journal of biological chemistry. 2014;289(2):1092-105.
8. Ouakad M, Vanaerschot M, Rijal S, Sundar S, Speybroeck N, Kestens L, et al. Increased metacyclogenesis of antimony-resistant Leishmania donovani clinical lines. Parasitology. 2011;138(11):1392-9.
9. Prus E, Fibach E. Flow cytometry measurement of the labile iron pool in human hematopoietic cells. Cytometry A. 2008;73(1):22-7.
10. Riedelberger M, Penninger P, Tscherner M, Seifert M, Jenull S, Brunnhofer C, et al. Type I Interferon Response Dysregulates Host Iron Homeostasis and Enhances Candida glabrata Infection. Cell host & microbe. 2020;27(3):454-66 e8.
11. Tenopoulou M, Kurz T, Doulias PT, Galaris D, Brunk UT. Does the calcein-AM method assay the total cellular 'labile iron pool' or only a fraction of it? The Biochemical journal. 2007;403(2):261-6.
12. Vinet AF, Fukuda M, Turco SJ, Descoteaux A. The Leishmania donovani lipophosphoglycan excludes the vesicular proton-ATPase from phagosomes by impairing the recruitment of synaptotagmin V. PLoS pathogens. 2009;5(10):e1000628.
13. Verma JK, Rastogi R, Mukhopadhyay A. Leishmania donovani resides in modified early endosomes by upregulating Rab5a expression via the downregulation of miR-494. PLoS pathogens. 2017;13(6):e1006459.
14. Riemer J, Hoepken HH, Czerwinska H, Robinson SR, Dringen R. Colorimetric ferrozine-based assay for the quantitation of iron in cultured cells. Anal Biochem. 2004;331(2):370-5.
15. Banerjee S, Datta R. Leishmania infection triggers hepcidin-mediated proteasomal degradation of Nramp1 to increase phagolysosomal iron availability. Cellular microbiology. 2020;22(12):e13253.
16. Banerjee S, Datta R. Localized Leishmania major infection disrupts systemic iron homeostasis that can be controlled by oral iron supplementation. The Journal of biological chemistry. 2023;299(8):105064.
17. Mukherjee B, Mukhopadhyay R, Bannerjee B, Chowdhury S, Mukherjee S, Naskar K, et al. Antimony-resistant but not antimony-sensitive Leishmania donovani up-regulates host IL-10 to overexpress multidrug-resistant protein 1. Proceedings of the National Academy of Sciences of the United States of America. 2013;110(7):E575-82.
18. Kang WS, Kwon JS, Kim HB, Jeong HY, Kang HJ, Jeong MH, et al. A macrophage-specific synthetic promoter for therapeutic application of adiponectin. Gene Ther. 2014;21(4):353-62.
