# Supplementary material for: Oxidative stress-driven enhanced iron production and scavenging through Ferroportin reorientation worsens anemia in antimony-resistant Leishmania donovani infection
Source: PLoS Pathog. 2025 Jan 31;21(1):e1012858. doi: 10.1371/journal.ppat.1012858 (PMC11785346; doi:10.1371/journal.ppat.1012858)
Supplement: Attachment — Submitted filename: 17.10.24_PPATHOGENS-D-24-00843_Authors Response.pdf [file ppat.1012858.s012.pdf]

To
The Editors
Plos Pathogens

Dear Editors,

Firstly, we would like to express our gratitude for allowing us to revise the manuscript **PPATHOGENS-D-24-00843**. We would also like to thank the reviewers for their effort in provide their assessment of this manuscript. We have carefully gone through the decision letter and we noted that the decision not to accept the manuscript is primarily due to several concerns raised by Reviewer 1.

In the rebuttal we have addressed all these concerns individually by providing a point-by-point responses to every single concern raised by both the reviewers. We have also conducted the additional animal experiment as suggested by the Reviewer 1, which we believe have make the conclusion of this work stronger. In view of these points, we strongly believe that this work would be considered for publication in the Plos Pathogens.

We look forward to hearing back from you.

Best Regards
Budhaditya

<u>Point by point response</u>

<u>Reviewer #1</u>: The manuscript contains significant flaws that undermine the validity and reliability of its conclusions. The main issues are outlined below. Given these critical issues, the manuscript's conclusions are not substantiated by the data presented. The inconsistencies, lack of essential data, and inadequate methodological details significantly diminish the credibility and scientific contribution of this manuscript. Therefore, the manuscript is recommended for rejection.

Response: We recognize that the reviewer has identified issues with our work. However, we believe that most of these comments do not raise significant concerns and are likely due to the reviewer either missing or misinterpreting our intended message, possibly due to the extensive amount of data. In the next paragraph, we have explained our reasoning and provided a detailed point-by-point response to all the concerns raised by the reviewer.

Part II – Major Issues: Key Experiments Required for Acceptance

Please use this section to detail the key new experiments or modifications of existing experiments that should be <u>absolutely</u> required to validate study conclusions. Generally, there should be no more than 3 such required experiments or major modifications for a "Major Revision" recommendation. If more than 3 experiments are necessary to validate the study conclusions, then you are encouraged to recommend "Reject".

Reviewer #1:

Comment 1: Lack of Supporting Data:

1) Organ-Parasite Load: The authors conclude about "organ-parasite" load in the 1st Results section without presenting any data on data. This omission makes the conclusion unsupported. Organ Weight: Similarly, the discussion on significant differences in organ weight is not backed by any data on organ weight, leading to unsupported claims.

Author Response: There are two issues raised by the reviewer that we would like to address one by one.

Organ-Parasite Load: We want to clarify that we have provided the data on organ parasite load utilizing two different experimental approaches in the **Original Submission**. **Figure 1C.i.** and **1C. iii.**, of the **Original submission** represents the data related to organ parasite load. **Figure 1C.i.** measures the expression of amastin in the spleen of infected mice and this is a well-accepted method reported by multiple groups to quantify organ parasite load (de Paiva, Grazielle-Silva 2015, PLoS pathogens ; Roy, Sarkar 2022, Parasitology). Apart from this, the organ-parasite load is also determined by Leishman-Donovan units (LDU), which is calculated as amastigote per nucleated cell * organ weight in mg (Khanra, Sarraf 2017, Scientific reports ; Mukherjee, Mukherjee 2012, PLoS neglected tropical diseases). We have also provided LDU values for all the experimental groups in **Figure 1C.iii.**

In the **Revised MS**, as per **Comment 2** by the reviewer, we have repeated the *in-vivo* competition assay with GFP-susceptible strain and RFP-resistant strain, and thus we have to replace the existing data for amastin and LDU with the new data set (**Figure R1, and R2, representing Figure 1B.ii., and 1C in the Revised MS**). Please note even in this repeat experiment we observed a similar trend as we have observed in *in-vivo* competition assay performed with GFP-susceptible and untagged-resistant strain in

the **Original Submission**. This confirms beyond any reasonable doubt that clinical strains with unresponsiveness towards antimony (LD-R) outcompete antimony susceptible strains (LD-S) in *in-vivo* murine infection.

Figure R1

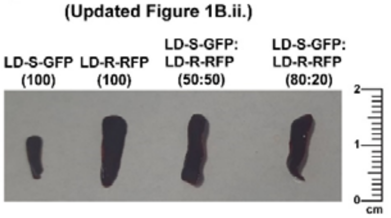

<u>Figure R1 representing Figure 1B.ii. in the revised MS:</u> Representative spleen image of infected-spleen 28 days pi from mice representing Groups: 1-4 (from left to right) with a scale bar (cm) at the right for differential comparison.

Figure R2

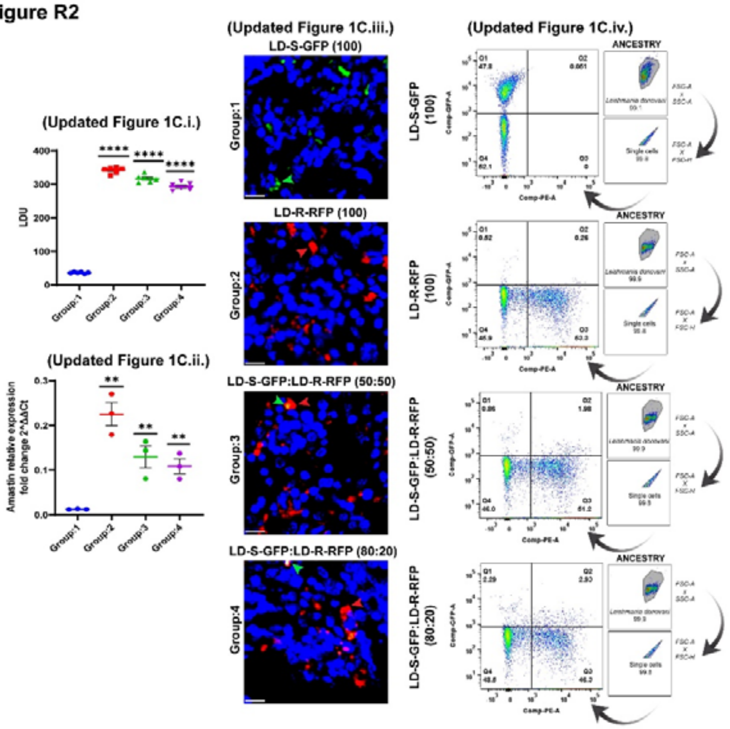

<u>Figure R2 representing Figure 1C in the revised MS: (1C.i.)</u> Graph showing LDU in each experimental set (Groups: 1-4). N=3 for each group in duplicate. **(1C.ii.)** Relative amastin expression fold change ($2^{\Delta\Delta Ct}$) in the infected-spleen isolated 28 days pi from Groups:1-4 (N=3 for each group) was determined from qRT-PCR depicting the amastigote load in each set with respect to murine β-actin. **<u>(1C.iii.)</u>** Confocal images of the cryosectioned spleen sample 28 days pi for experimental groups: 1-4 representing the LD-S-GFP and LD-R-RFP amastigote load (green dots marked in green arrow and red dots marked in red arrow) in each splenic sample. Scale bars indicate 20µm. **<u>(1C.iv.)</u>** % of the GFP-positive population enumerated from flow cytometry to denote the load of LD-S-GFP in comparison to LD-R in experimental Groups: 1-4. The right-most panel shows the ancestry of each analysis. Each experiment was performed in triplicates and graphs are represented as Mean with SEM. P ≤ 0.0001 is marked as ****, and P ≤ 0.01 is marked as **.

Organ Weight: Regarding the reviewer's comment on "organ weight", it has been clearly mentioned in lines 552 of the **Original Submission** that spleen weight was measured and used to calculate the LDU (LDU= amastigote per nucleated cell * organ weight in mg) for each experimental group. Since we have provided the LDU in the original MS, we didn't provide spleen weight separately, as it would have not added anything substantially new. However, as the reviewers have suggested we are providing the organ weight of individual spleen isolated from repeat experimental sets of infected mice as per **Comment 2 (Figure R3)**. We would also like the reviewer to note that like our **Original submission** we have also provided visual representative images of the individual spleens **(Figure R1)** from each repeat experimental groups in **Figure 1B.ii.** in the **Revised MS** reflecting similar variation in their sizes

as in the **Original MS (Figure 1B.ii.** in the **Original MS)**, which again should substantiate their difference in weight. With all these data, we believe that we have convincingly pointed out that we haven't omitted any critical results that make our claim unsupported in any possible logical way. Please note in the **Revised MS** as we have repeated this experiment with GFP-susceptible strain and RFP-resistant strain as per reviewer's comment 2, the individual spleen weights obtained from this experiment has been used to calculate LDU, and the mean spleen weights are mentioned in the **Revised MS**. All these information has been mentioned in the result section of the **Revised MS** lines 138-143 as "Firstly, a significantly larger infected spleen with increased weight was observed in Group:2 (~120.566mg), Group:3 (~118.266mg), and Group:4 (~116.633mg) mice as compared to Group:1 (~32.766mg) mice spleen **(Figure 1.B.ii.)** which was used for calculating LDU. In Group:2, Group:3 and Group:4 infected-spleen LDU index was significantly higher as compared to Group:1 infected spleen **(Figure 1C.i.)**."

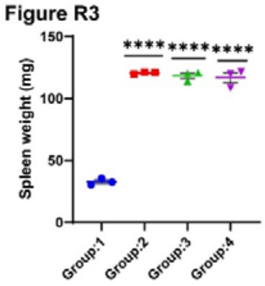

Figure R3: Bar graph representing infected mice spleen weight of different experimental sets (Group:1-4) expressed in mg (N=3). Group:1 representing LD-S-GFP, Group:2 representing LD-R, Group:3 representing LD-S-GFP: LD-R in 50:50 ratio, Group:4 representing LD-S-GFP: LD-R in 80:20 ratio. Group:2, Group:3, and Group:4 (****, P ≤ 0.0001) shows a significant increase in spleen weight as compared to Group:1.

Comment 2: Inconsistent Methodology:

In Vitro vs. In Vivo Comparisons: For in vitro infection, a GFP susceptible strain and a RFP resistant strain are compared, but for in vivo experiments, a GFP susceptible strain is compared with a non-fluorescent resistant strain. The methodology should be consistent, and the same tagged resistant strain should be used in both in vitro and in vivo experiments to track both strains in microscopy and cytometry experiments.

Response: We understand and acknowledge this comment raised by the reviewer. However, we would like to clarify that it was an informed choice on our part not to use RFP-resistant strain in *in-vivo* competition experiments for a logical reason as follows:

We argue that since resistant strains show aggressive proliferation *in-vitro* (**Figure 1A.iv. in the Original and Revised submission**), we assume that the RFP-positive resistant parasite strain will possibly outcompete the GFP-positive susceptible population in infected mice, thus making it difficult to track the GFP-positive population (even if it exists in a low level) from within a larger RFP-resistant population in competition experimental sets (Group:3 and Group:4). Hence, we chose an untagged resistant strain to make sure we could better track the GFP signal, even if it is severely diminishment in the *in-vivo* competition assay.

However, we acknowledge the concern raised by the reviewer to maintain consistency in selecting tagged sensitive and resistant strains in in-vitro and in-vivo experimental setups. Hence, we have repeated this experiment with GFP-susceptible strain and RFP-resistant strain, and the revised results have been presented as **Figure R1 and R2 (Figure 1B** and **1C** in the **Revised MS)**. The results have been explained in line numbers 135-150 and 154-167 in the **Revised MS**. This result is consistent with the previous result obtained by competing GFP-tagged LD-S with untagged LD-R strain.

Comment 3: Missing Data and Results:

No results are presented for the description on lines 156-158.

The data does not show differences among Groups 2, 3, and 4, contrary to the discussion on lines 143-148.
Essential data on parasite proliferation after 48 and 72 hours of in vitro infection and in vivo organ-parasite load, are missing.

Response: There are 3 points raised by the reviewer here, we have answer them one by one.

A. No results are presented for the description on lines 156-158.

Response: Lines 156-158 in the **Original Submission** reads as, "Notably, it took approximately 11-13 days for Group:1, 5-7 days for Group:2, and 7-10 days for Group:3 and Group:4, for respective LD-promastigotes to emerge from the infected macerated spleens."
We didn't feel that it was necessary to plot the number of days in the graph as this has been already clearly mentioned in the results.
Please note in the **Revised MS,** line numbers 159-161 this statement has been updated as "Notably, it took approximately 10-13 days for Group:1, 5-7 days for Group:2, and 6-8 days for Group:3 and Group:4, for respective LD-promastigotes to emerge from the infected macerated spleens." as per result obtained from the conformational experiment performed with GFP-susceptible strain and RFP-resistant strain.
Also, for the reviewer's convenience, we have plotted the days it took for amastigote to promastigote differentiation from macerated infected spleen for respective new experimental sets for individual mice in each group (**Figure R4**).

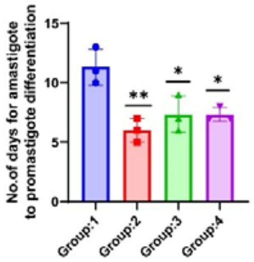

Figure R4: Bar graph showing the number of days it took for splenic amastigotes to differentiate into promastigotes in the culture for each experimental group (N=3). Group:2 showed significantly less number of days (**, P ≤ 0.01) followed by Group:3 and Group:4 (*, P ≤ 0.05) for amastigote-promastigote differentiation as compared to Group:1.

B. The data does not show differences among Groups 2, 3, and 4, contrary to the discussion on lines 143-148.

Response: Lines 143-148, in the **Original Submission,** reads as "This observation was in concurrence with amastin expression showing the highest splenic amastigote load for Group:2-infected-mice, followed by Group:3 and Group:4, all of which are significantly higher compared to Group:1-infected-mice **(Figure 1C.i.)**. Fluorescence microscopy of the splenic sections **(Figure 1C.ii.)** revealed GFP-positive amastigotes around the splenic MΦs in Group:1 whereas in Group:2, there was no signal of GFP as anticipated."
We would like to clarify that in the **Original Submission,** we have not claimed that there were significant differences in amastin expression within Group:2, Group:3, and Group:4 infected mice. We have clearly stated in the **Figure 1C.i.,** that amastigote load is highest for Group:2 infected mice, followed by Group:3 and Group:4 infected mice. And all these groups show significantly higher amastigote load compared to Group:1 infected mice (*, P ≤ 0.05). Similarly, for **Figure 1C.iii.**, we observed that LDU was highest for Group:2 followed by Group:3 and Group:4, all of which are significantly higher than Group:1(****, P ≤ 0.0001). Furthermore, the fluorescence microscopy images of **Figure 1C.ii.** also clearly presented this difference with significant reduction of GFP positive signal in Group:3 and Group:4-infected mice. The point of this result is to show that whether Group:3 and Group:4 mice bearing mixed infection shows similar infection load as Group: 2 LD-R infected mice which we believe this data effectively conveys.

We apologize for the confusion. We have updated this result section in the **Revised MS** as per the data obtained from repeat experimental sets as represented in **Figure R2** (**representing Figure 1C in the revised MS**). The revised sentence (line number 135-150) now reads as "Further, *in-vivo* experiments in BALB/c mice infected with an equal number of sorted metacyclic *i.e.* either with LD-S-GFP (Group:1), with LD-R-RFP (Group:2), with a mixture of LD-S-GFP: LD-R-RFP in 50:50 ratio (Group:3), or with LD-S-GFP: LD-R in 80:20 ratio (Group:4) corroborated these findings. Firstly, a significantly larger infected spleen with increased weight was observed in Group:2 (~120.566mg), Group:3 (~118.266mg), and Group:4 (~116.633mg) mice as compared to Group:1 (~32.766mg) mice spleen **(Figure 1.B.ii.)** which was used for calculating LDU. In Group:2, Group:3 and Group:4 infected-spleen LDU index was significantly higher as compared to Group:1 infected spleen **(Figure 1C.i.)** indicating towards higher infection-load as compared to Group:1. This observation was in concurrence with amastin expression showing the highest splenic amastigote load for Group:2-infected-mice, followed by Group:3 and Group:4, all of which are significantly higher compared to Group:1-infected-mice **(Figure 1C.ii.)**. Fluorescence microscopy of the splenic sections **(Figure 1C.ii.)** revealed RFP-positive LD-R amastigotes outcompeted GFP-positive LD-S amastigotes around the splenic MΦs in Group:3 and Group:4 infected spleen. Group: 1 (LD-S-GFP) and Group:2 (LD-R-RFP) infected spleen were used as infected control where also it was evident that LD-R-RFP shows higher amastigote load as compared to LD-S-GFP **(Figure 1C.iii.)**.

Figure R2

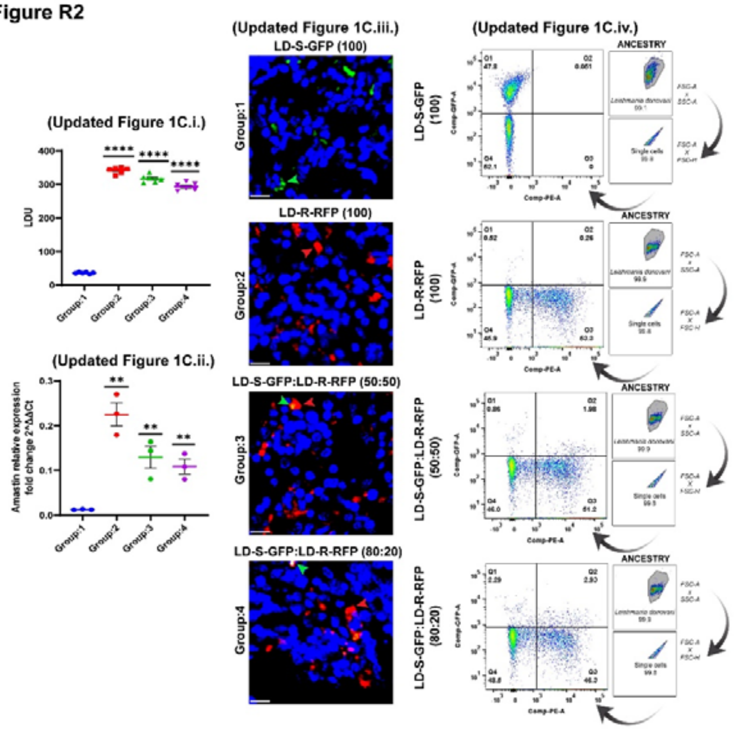

Figure R2 representing Figure 1C in the revised MS: (1C.i.) Graph showing LDU in each experimental set (Groups: 1-4). N=3 for each group in duplicate. **(1C.ii.)** Relative amastin expression fold change (2^ΔΔCt) in the infected-spleen isolated 28 days pi from Groups:1-4 (N=3 for each group) was determined from qRT-PCR depicting the amastigote load in each set with respect to murine β-actin. **(1C.iii.)** Confocal images of the cryosectioned spleen sample 28 days pi for experimental groups: 1-4 representing the LD-S-GFP and LD-R-RFP amastigote load (green dots marked in green arrow and red dots marked in red arrow) in each splenic sample. Scale bars indicate 20µm. **(1C.iv.)** % of the GFP-positive population enumerated from flow cytometry to denote the load of LD-S-GFP in comparison to LD-R in experimental Groups: 1-4. The right-most panel shows the ancestry of each analysis. Each experiment was performed in triplicates and graphs are represented as Mean with SEM. P ≤ 0.0001 is marked as ****, and P ≤ 0.01 is marked as **.

C. Essential data on parasite proliferation after 48 and 72 hours of in vitro infection and in vivo organ-parasite load, are missing.

Response: In this entire study, we have clearly defined early time points as 4hrs post-infection and late time points as 24hrs post-infection for all the in-vitro experimental designs. We would like to clarify that the selection of these time points is deliberate, not random. We focused on these two time-points because the trend in amastigote proliferation differences between LD-S and LD-R observed at 24 hours, also persists at 48 and 72 hours, hence were not included in the **Original Submission**. However, as suggested by the reviewer we are providing this data (**Figure R5**) and have included this data as **Figure S1B.i.** and **S1B.iii.** in the **Revised MS Supplementary section**.

Regarding the *in-vivo* organ-parasite load, in the **Original submission** lines 544-545 of the materials and methods section, it is clearly mentioned, "mice were sacrificed at 28days pi (for splenic amastigote load) or 24weeks pi (for establishing anemia model) as per experimental requirements." It is very well-established that splenic parasite burden in LD infected mice starts appearing around 14-28days pi (Cotterell, Engwerda 2000, Infection and immunity ; Lang, Ave 2000, Cellular microbiology). Hence, it is not possible to provide the data on 48-72hrs infection to determine *in-vivo* organ parasite load as suggested by the reviewer. We firmly believe that the reviewer will now be convinced that there is no 'missing data' described in the results section without supporting evidence.

Figure R5

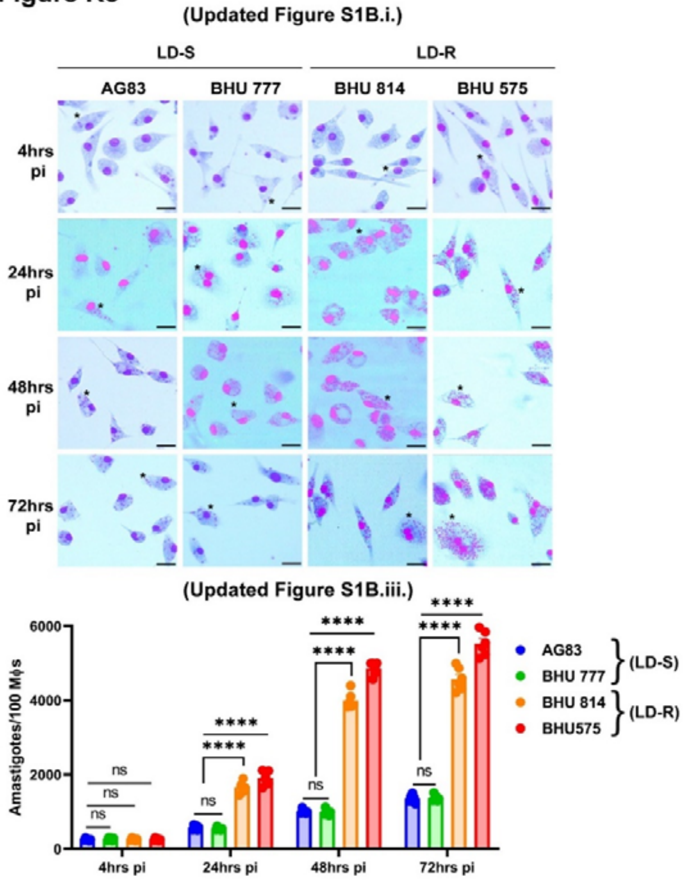

Figure R5: Antimony-resistant phenotype coincides with higher parasite burden if metacyclogenesis is kept as a constant factor. (S1B.i.) Giemsa-stained images of an equal number of AG83, BHU777 (representative LD-S) and BHU814, and BHU575 (representative LD-R) metacyclic promastigote infected-MΦs at 4hrs,24hrs, 48hrs, and 72hrs pi. One representative small nucleus of LD has been marked in (*) to show the infected-MΦs. **(S1B.iii.)** Amastigotes/100 MΦs at 4hrs, 24hrs, 48hrs, and 72hrs pi were calculated taking different fields, and the data represents the Mean ± SEM of three independent experiments. **** denotes P ≤ 0.0001 and ns denotes P > 0.05.

Comment 4: Poor Image Quality and Inadequate Controls: Confocal images in Fig 1C.ii and FigS1D.i are of poor resolution and do not support any conclusions.
Figure 2A lacks scale bars and controls, making it difficult to interpret the results accurately.

Response: We would clarify each of this concern in a point-by-point manner.

A. Confocal images in Fig 1C. ii and FigS1D.i are of poor resolution and do not support any conclusions.

Response: We would like to point out that there are multiple other confocal images which has been provided in the **Original MS (Now represented as Figure 2A.i., 2B.i., 3D.ii.a., 4A.i., 4Bi., 4C.i.a., 4D.i., 4D.ii., 4E.i.a., 5A.i., 5A.iii., 5B.i.a., 6B.ii., 6C, S3A.i., S3B, S3C, S3D, S4A.ii.,** and **SB. ii. a in the Revised MS)** out of which reviewer have pointed out only two images as poor-quality images, and reason behind this is entirely technical as we have explained in the next paragraph.
Both **Figure 1C. ii** and **S1D.i.** in the **Original Submission** are representative fluorescence images of the whole spleen macerate of the infected murine spleen in lower magnification (20X). This was performed specifically to capture the GFP-positive amastigote signal in a wide region of view. We have clearly mentioned these in the Figure legends of the respective figures lines 829-831 of the **Original Submission** which reads as "Confocal images, of the macerated spleen sample 28 days pi for experimental groups: 1-4 representing the GFP-amastigote load (small green dots) in each splenic sample. Scale bars indicate 20μm.**".** Since these two images represent the whole splenic macerates with low magnification, it is understandable that these two particular images are of not as superior quality as compared to all the other images provided in the MS. However, we believe these images still clearly represent the differential GFP expression among different experimental groups and convey the required message effectively. We hereby have included the enlarged version of those images to show this.

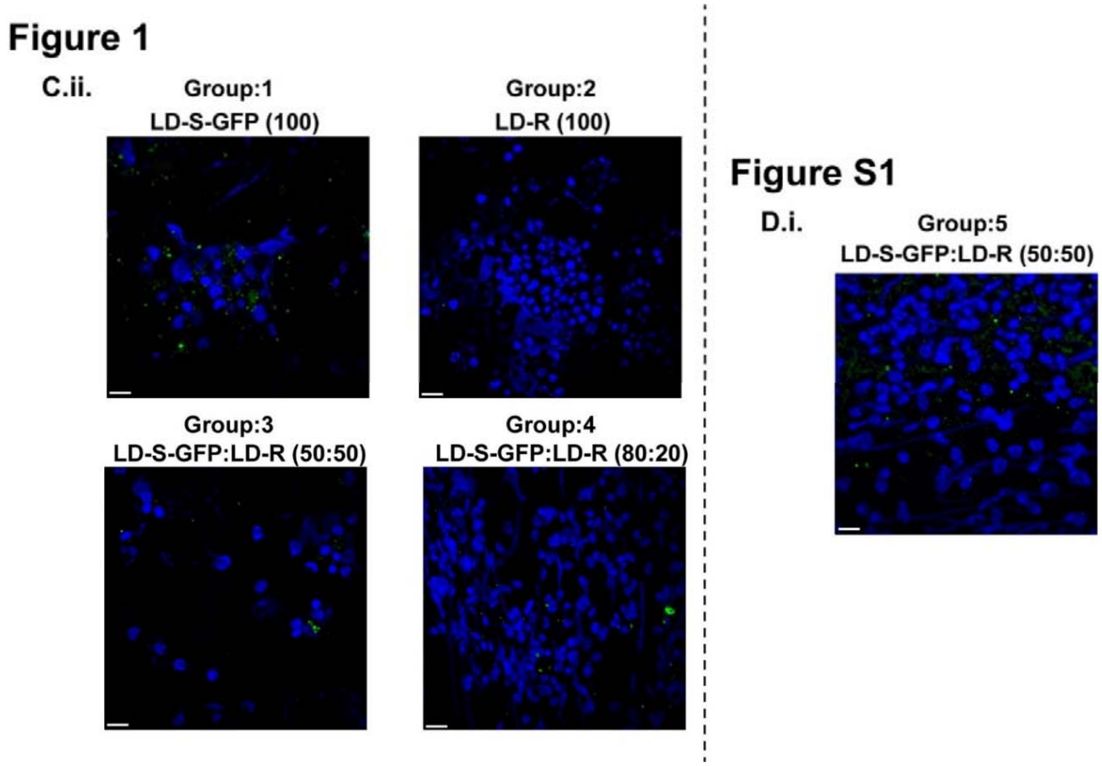

Figure 1C.ii. and Figure S1D.i.: Confocal images of the macerated whole spleen sample 28 days pi for experimental groups: 1-4 and 5 respectively representing the GFP-amastigote load (small green dots) in each splenic sample. Scale bars indicate 20μm.

Moreover, in the **Revised MS**, to acknowledge this concern, in the repeat experiment performed with **GFP-susceptible and RFP-resistant strain**, we have used cryosectioned spleen of infected mice to acquire better resolution confocal images as represented in **Figure R2** which we have included in the revised MS as **Figure 1C.iii.** We hope reviewer will appreciate our effort in addressing this technical issue. Also, we would like to point out that like our **Original submission**, the conclusion of these images are also supported independently by multiple other results in the **Revised MS** (**Figure 1B.ii.**, **1C.i.**, **1C.ii** and **1C. iv.)** all of which have independently provided evidence to support the conclusion that LD-R outcompetes LD-S in competitive mixed infection.

Figure R2

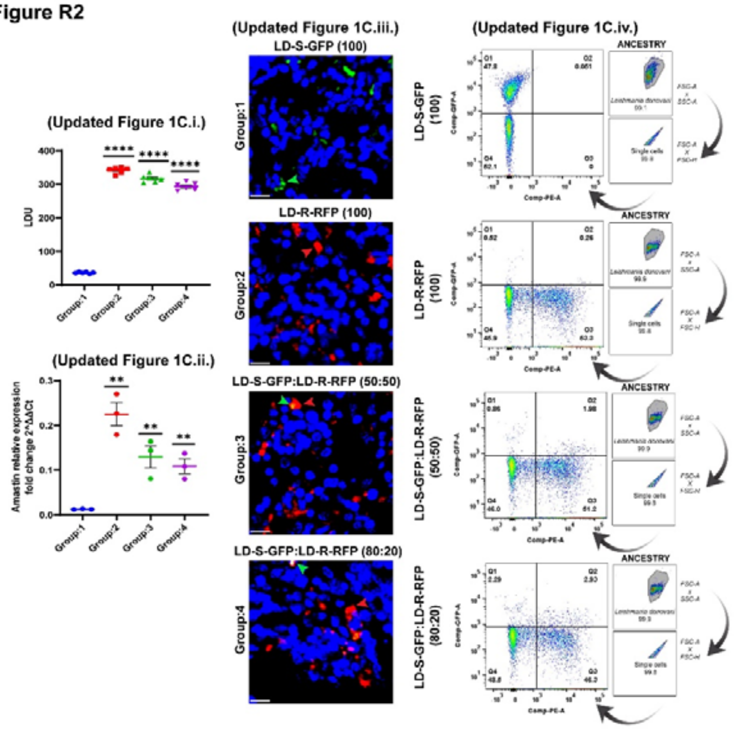

Figure R2 representing Figure 1C in the revised MS: (1C.i.) Bar graph showing LDU in each experimental set (Groups: 1-4). N=3 for each group in duplicate. **(1C.ii.)** Relative amastin expression fold change (2^ΔΔCt) in the infected-spleen isolated 28 days pi from Groups:1-4 (N=3 for each group) was determined from qRT-PCR depicting the amastigote load in each set with respect to murine β-actin. **(1C.iii.)** Confocal images of the cryosectioned spleen sample 28 days pi for experimental groups: 1-4 representing the LD-S-GFP and LD-R-RFP amastigote load (green dots marked in green arrow and red dots marked in red arrow) in each splenic sample. Scale bars indicate 20μm. **(1C.iv.)** % of the GFP-positive population enumerated from flow cytometry to denote the load of LD-S-GFP in comparison to LD-R in experimental Groups: 1-4. The right-most panel shows the ancestry of each analysis. Each experiment was performed in triplicates and graphs are represented as Mean with SEM. $P \leq 0.0001$ is marked as ****, and $P \leq 0.01$ is marked as **.

B. Figure 2A lacks scale bars and controls, making it difficult to interpret the results accurately.

Response: Figure 2A in the **Original Submission** represents two figures (**Figure 2A.i. and 2A.ii.**). We believe the comment regarding the scale bar is related to **Figure 2A.i.** We would like to thank the reviewer for pointing this out, actually if observed closely, **Figure 2A.i.** has scale bars (in black) and has been mentioned in the figure legend (please see the zoomed section). But we agree this is probably not visible to the reviewer as well as readers and we apologize for not making it more prominent. Hence, we are also adding it outside the image for better visibility (**Figure R6A represented as Figure 2A.i. in the Revised MS**).

Figure 2

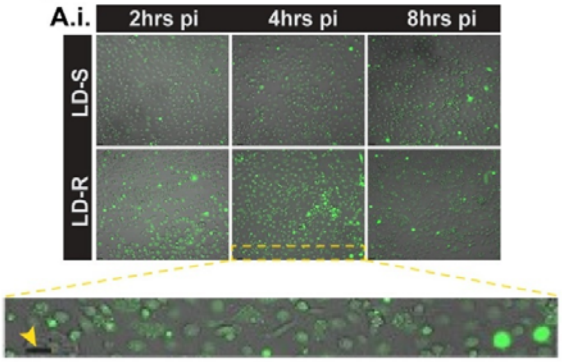

Figure 2: (Ai.) 20X-magnification live-cell confocal images of H_2DCFDA-stained (green) LD-S and LD-R infected-MΦs at early hours (2, 4, and 8hrs pi) with zoomed area (demarcated in yellow dotted area) showing scale bar of 20μm (marked in yellow arrow).

Regarding the '**controls**' mentioned by the reviewer, we would like to point out that as **Figure 2A** specifically compares the differential ROS induction between LD-R and LD-S infected macrophages at multiple time points, we believe it was not necessary to include additional uninfected control in this figure to reduce the figure burden, although we had all experimental sets all along and as suggested by the reviewer, we are including this in the **Revised MS**. Similarly, for flow cytometry estimation of the DCF-FITC-A positive population (**Figure 2A.ii.**), we have used uninfected unstained peritoneal macrophage control to gate the macrophage population and 100μM H_2O_2-treated macrophage positive control (Srivastav, Basu Ball 2014, The Journal of biological chemistry) for gating the DCF-FITC-A positive population, we have included these controls in the **Revised MS** (**Figure R6A and R6B** and have updated **Figure 2A.i.** and **2A.ii.** respectively in the **Revised MS**).

Figure R6

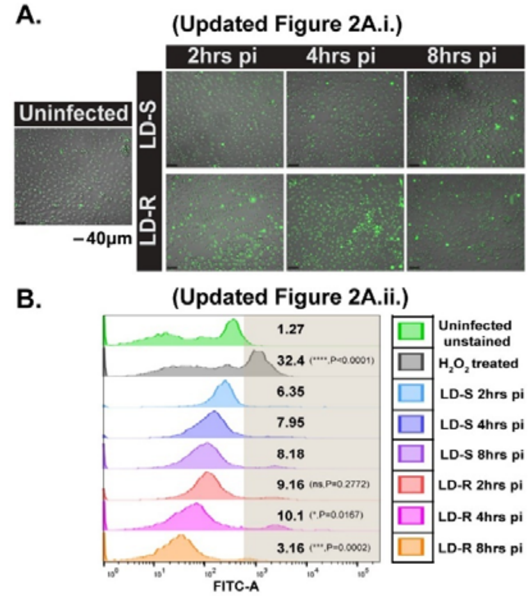

<u>Figure R6A. represented as Figure 2A.i. in the Revised MS:</u> 20X-magnification live-cell confocal images of H_2DCFDA-stained (green) LD-S and LD-R infected-MΦs at early hours (2, 4, and 8hrs pi). The black scale bar outside the image represents 40μm. **<u>Figure R6B. represented as Figure 2A.ii. in the Revised MS:</u>** Representative Flow cytometry images of half offset histogram plot representing live-cell quantification of DCF fluorescence in FITC-A filter in LD-S and LD-R infected MΦs at 2hrs, 4hrs, and 8hrs pi. Uninfected unstained control was used for gating the macrophage population and 100μM H_2O_2 treated macrophage positive control (1hr treatment) was used to gate the DCF-positive population in the FITC-A channel. The grey area demarcates the DCF-FITC-A-positive population. H_2O_2-treated macrophages showed significant ROS outbursts as compared to uninfected unstained control (****, $P \leq 0.0001$). LD-R 2hrs pi showed no significant change as compared to LD-S 2hrs pi (ns, $P > 0.05$), while at 4hrs pi, LD-R showed significant ROS outburst compared to LD-S infection (*, $P \leq 0.05$), and at 8hrs pi LD-R shows a significant drop in ROS level as compared to LD-S infection (***, $P \leq 0.001$), N=3 independent biological repeats.

Comment 5: Unsupported Conclusions:

ROS Levels: Conclusions on ROS levels differences between LD-S and LD-R strains are not supported by the presented data and statistical analysis (lines 168-171). Ferritin Levels: Conclusions on Ferritin levels are not supported by the densitometry analysis of the western blot in Fig. 3B. Quantification of HO-1 levels and biological replicates for the

western blot analysis are also missing. Luciferase Activity: The conclusion that luciferase activity modulation is exclusive to LD-R is not accurate, as activity modulation is also observed in LD-S.

Response: Again, there are 3 concerns and we have addressed individual concerns one by one.

A. ROS Levels: Conclusions on ROS levels differences between LD-S and LD-R strains are not supported by the presented data and statistical analysis (lines 168-171).

Response: Line 168-171 in the **Original MS** reads as, "However, LD-R-infected-MΦs exhibited higher ROS levels at 2hrs and 4hrs pi compared to LD-S-infected-MΦs, which flattened out by 8hrs pi **(Figure 2A.i. and A.ii., S1E)**. However, incubation of MΦs with killed-LD-R failed to generate substantial ROS, indicating that replicating LD-R is required for ROS generation **(Figure S1E)**."

We have presented the Flow data as a histogram plot and not as a bar graph for better representation but we agree we should have included the p-value. We have rectified this mistake and have included the same and updated it in **Figure R6B represented as Figure 2A.ii.** in the **Revised MS** which clearly supports the conclusion on the ROS levels.

We would also like to bring to the reviewer's attention, in addition to Confocal and Flow cytometer-based quantifications, we have also performed Fluorescence plate reader-based quantification of ROS, which is presented in **Figure S1E** of the **Original Submission**, the statistical significance of both these results are provided which again clearly supports the conclusion of the sentence (168-171) referred by the reviewer. Apart from this, statistical analysis for the ROS measurement represented in Figures S1F and S1G further strengthens our point. With these evidences from multiple independent experiments, we strongly feel that we will be able to convince the reviewer.

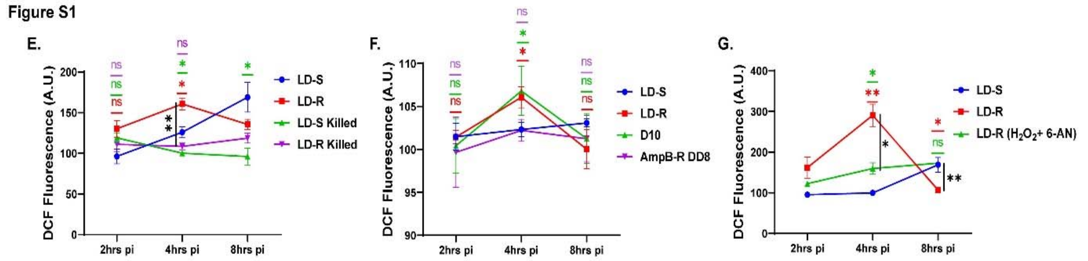

Figure S1. ROS level monitoring (E.) DCF-fluorescence quantification of LD-S, LD-R, heat-killed-LD-S, and heat-killed-LD-R infected-MΦs at early hours. Heat-killed LD-R experimental sets at 4hrs pi significantly fail to generate ROS as compared to LD-R infected-MΦs at 4hrs pi (**, $P \leq 0.01$). **(F.)** DCF-fluorescence quantification of MΦs infected with LD-S, LD-R, D10, and AmpB-R DD8 **(G.)** DCF-fluorescence quantification of MΦs infected with LD-S or LD-R, or NADPH^{exh}-LD-R by H_2O_2+6-AN treatment. LD-R (H_2O_2+6-AN) significantly failed to generate ROS as compared to LD-R infected-MΦs at 4hrs pi (*, $P \leq 0.05$). At 8hrs, LD-R infected- MΦs shows a significant drop in ROS level compared to LD-R (H_2O_2+6-AN) (**, $P \leq 0.01$), which remains unchanged like 4hrs pi. P-value summary is color-coded corresponding to the color of individual experimental sets and all the analysis is performed compared to LD-S infected MΦs at individual time points except for the one in black in Figure S1E and S1G. ** denotes $P \leq 0.01$, * denotes $P \leq 0.05$, and ns denotes $P > 0.05$.

B. Ferritin levels: Conclusions on Ferritin levels are not supported by the densitometry analysis of the western blot in Fig. 3B. Quantification of HO-1 levels and biological replicates for the western blot analysis are also missing.

Response: We are providing Densitometry analysis and the 2 additional biological replicates of Ferritin and HO-1 apart from the one already provided in Figure 3B of the **Original Submission** as requested by the reviewer (**Figure R7**). These densitometry with statistical analysis are now included in the **Revised MS** as **Figure 3B ii.a. and Figure 3B.ii.b.** and have been mentioned in the Figure legends.

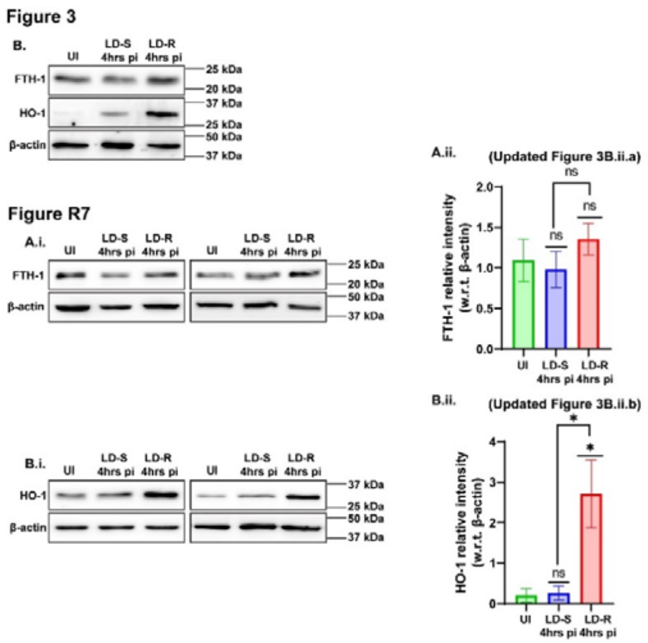

Figure R7 represented as Figure 3B: Western blots of Ferritin (FTH-1) and Heme oxygenase 1 (HO-1) in triplicates (**Figure 3B**, **R7A.i.** and **R7B.i.**) and the representative bar graphs of relative intensity fold change with respect to β-actin (Figure R5A.ii. and R5B.ii. respectively). **(R7A.ii. represented as Figure 3B.ii.a.)** No significant change in FTH-1 was observed in any experimental conditions (ns, $P > 0.05$). **(R7B.ii. represented as Figure 3B.ii.b.)** Significant upregulation of relative intensity of HO-1 is observed in LD-R 4hrs pi as compared to both UI and LD-R 24hrs pi (*, $P \leq 0.05$) whereas no significant change is observed in LD-S 4hrs pi as compared to UI (ns, $P > 0.05$).

We would also like to mention that we have included the Densitometry analysis for all other western blots provided in the MS along with their statistical analysis in the **Revised MS** to convince the reviewer. The combined **Figure R10 and Figure RS1 represents those** Densitometry analysis. All the Densitometry plots are now presented for respective western blot images in the **Revised MS**, such as **Figure 3D.i.b.i.** and **3D.i.b.ii.** for p50 and cRel respectively; **Figure S3A.ii.b.**, **S3B**, and **S3F** for CD71, Fpn, and NRAMP1 respectively; **Figure S4A.i.b.** and **S4B.i.b.** for p62, and NRF2 respectively; **Figure 5B.ii.b.** for SIRPα; **Figure 6A.ii.**, and **6B.i.b.** for ADAM10 and Furin respectively.

C. Luciferase Activity: The conclusion that luciferase activity modulation is exclusive to LD-R is not accurate, as activity modulation is also observed in LD-S.

Response: We completely agree with the reviewer on this. Some level of changes although minor is also noticed for LD-S infection (**Figure 3E in the Original and Revised MS**). We regret that we have missed this and thank the reviewer for pointing this out. We have addressed this by modifying the conclusion as suggested by the reviewer and have included this in the results section lines 251-257 of the **Revised MS**. **"**Finally, Luciferase assay with wild-type, or truncated promoter constructs: Site $A^{-/-}$ (-4/-635) and Site $B^{-/-}$ (-636/-1377) in the presence of LD-R and LD-S infection, further confirmed specific interaction of p50/c-Rel with Site A of HO-1 promoter resulting in its specific upregulating in LD-R-infected MΦs **(Figure 3E)**. Interestingly, some luciferase activity modulation in the HO-1 promoter construct in response to LD-S-infection as compared to uninfected control was observed, probably owing to the effect of some other transcription factors having binding sites within the HO-1 promoter."

Comment 6: Inadequate Statistical Analysis:

Statistical analyses for several conclusions, including anemia-related findings, are not presented. Specifically, the HCT differences between LD-S and LD-R are not statistically significant (t test done by myself).

Response: We have clearly mentioned the statistical analysis including the number of biological repeats and *p*-value for all the results in respective figure legends **in the Original Submission**. Regarding anemia-related findings, we have now added statistical analysis in **Figure 7A.iii.**. Additionally, we have also added a paragraph in the Materials and Nethod section to avoid any further doubts on statistical analysis as follows:

"**Statistical Analysis**

Each experiment was performed in triplicates, independently. The results are expressed as mean ± SEM (in some cases as ± SD as specifically mentioned in the figure legends). Statistical significance was calculated by performing an unpaired t-test in GraphPad Prism 8.0.2, and a P-value <0.05 was considered statistically significant. Values were considered most significant (represented as: ****) if P ≤ 0.0001, extremely significant (represented as: ***) if P ≤ 0.001, very significant (represented as: **) if P ≤ 0.01, or significant (represented as *) if P ≤ 0.05; and non-significant (represented as: ns) if P > 0.05."

Figure 7
A.iii.

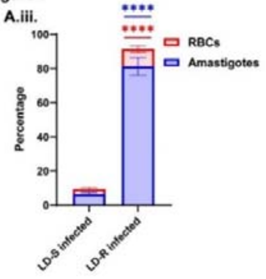

Figure 7: (A.iii.) Stacked bar plots showing % of amastigotes and RBC engulfed by erythrophagocytes in LD-S and LD-R infected-spleen. LD-R infected spleen shows significantly higher RBCs engulfed and amastigote burden as compared to LD-S infected spleen at 24weeks pi (****, P ≤ 0.0001).

Regarding the specific concern raised by the reviewer for the significance of the HCT difference between LD-S and LD-R infected mice, we would like to mention that it has been calculated using GraphPad Prism software *v* 8.0.2. GraphPad Prism is a well-accepted software for performing statistical analysis and is almost used by all researchers in the field. The P value comparing HCT between LD-S and LD-R was 0.0487, which is significant (please see the attached screenshot). We are not sure how without raw data points, the reviewer was able to calculate the statistical significance but we are highly confident about our statistical analysis for all our data and will be willing to share all these files if requested.

Unpaired t test	
Table Analyzed	HCT
Column C	LD-R infection
vs.	vs.
Column B	LD-S infection
**Unpaired t test**	
P value	0.0487
P value summary	*
Significantly different (P < 0.05)?	Yes
One- or two-tailed P value?	Two-tailed
t, df	t=2.803, df=4
**How big is the difference?**	
Mean of column B	35.37
Mean of column C	31.67

Figure: Screenshot showing unpaired t-test analysis performed in GraphPad Prism for HCT in LD-R infected blood vs LD-S infected blood at 24weeks pi.

Further, we would like to elaborate that honouring the reviewer's comments of low statistical significance we have repeated the experiments with an additional 3 mice in each group making the total number of mice for each experimental set 6. We have now included the statistical significance analysis (p-value) for LD-R infected blood with respect to LD-S infected blood for each of these parameters in the figure

to avoid any further confusion (**Figure R8,** representing **Figure 7D.i.** in the **Revised MS**). This has now also been clearly mentioned in the figure legends of the revised MS.

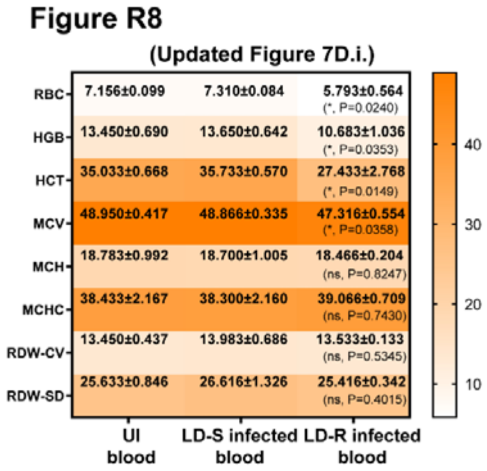

Figure R8 represented as 7D.i.: Heatmap showing the differential count of RBC-associated parameters in uninfected, LD-S, and LD-R infected-BALB/c mice. Data is represented as the Mean ± SEM where N=6.

Further, we have also provided additional confocal microscopy and Scanning Electron Microscopy data in this regard to support our conclusion. Splenic sections of these infected mice were incubated with DAB (which stains hemoglobin) and double stained with anti-F4/80 antibody (demarcating the red-pulp macrophages) and anti-CD47 antibody (marking the intact RBC membrane), to differentiate between phagocytosis of live intact and senescent RBCs in LD-S vs LD-R infected spleen, provided as **Figure R9** represented as **Figure 7B** in the **Revised MS**. This was further validated by high-resolution SEM images provided in **Figure R9** and represented as **Figure 7C** in the **Revised MS**. This has been mentioned in methods section lines 792-817 and results section lines 406-416 in the **Revised MS**.

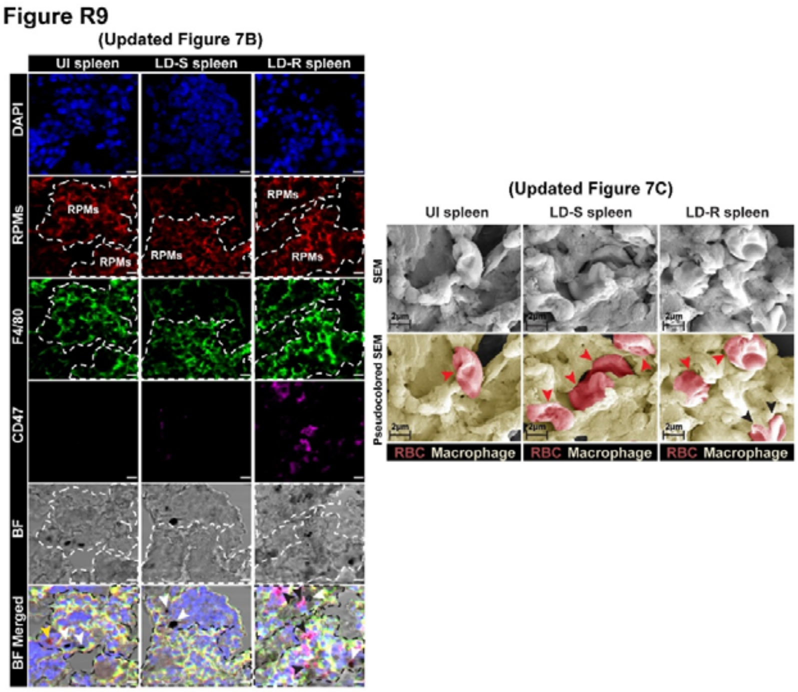

Figure R9: (7B.) Cryomicrotome sections of uninfected, LD-S, and LD-R 24weeks pi infected spleen showing autofluorescent red pulp macrophages (bright red, demarcated by a white dotted area) further stained by F4/80 (green). Live-intact RBCs were stained by CD47 antibody and ingested hemoglobin was stained by DAB. The black arrow denotes $CD47^{+}/DAB^{+}$ live intact RBCs, the white arrow denotes $CD47^{-}/DAB^{+}$ hemoglobin deposits, and the yellow arrow denotes $CD47^{+}/DAB^{-}$ RBC-ghosts in the merged BF image. Scale bars indicate 20µm. **(7C.)** SEM images (upper panel) of uninfected, LD-S and LD-R 24weeks pi infected murine spleen showing macrophages engulfing RBCs. The lower panel represents pseudocolored SEM where RBCs are colored in red and macrophages in yellow. The black arrow represents live RBCs with intact biconcave morphology and the red arrow represents senescent RBCs with deformed morphology.

Comment 6: Methodological Concerns:

The percentages of metacyclics shown in Fig 1Ai, Aii and Fig S1A are before or after sorting? Most published works report percentages below 40% before purification.

Calcein is not a reliable method for LIP quantification in this context (doi: 10.1042/BJ20061840, among others).
Many western blot-related conclusions are quantitative, yet the manuscript does not report how these quantifications were performed or the number of replicates.

Response: We are providing clarification for each of these points which should essentially clear out any doubts of the reviewer regarding all the methodological issues and should be able to convince him about stringency of all the methods used in this study.

A. The percentages of metacyclics shown in Fig 1Ai, Aii and Fig S1A are before or after sorting? Most published works report percentages below 40% before purification.

Response: We have no conflict against this comment of the reviewer. In fact, we also observed a higher percentage of metacyclic in resistant strains as compared to sensitive ones as previously reported by other groups (Ouakad, Vanaerschot 2011, Parasitology). That is why as mentioned in the materials and method section of the **Original MS**, "Metacyclic promastigotes were sorted using Beckman Coulter Cytoflex srt by gating *FSC-A* X *SSC-A* where FSC^{low} represents the metacyclic population and FSC^{high} represents the procyclic population." "Next, a portion of these sorted metacyclic promastigotes was further checked on the Flow cytometer capturing 20,000 events which is represented in **Figure 1A.i.**, **1A.ii.**, and **S1A** signifying uniform metacyclics between LD-S and LD-R for performing downstream experimental studies". We do agree with the reviewer that for the clarity of readers, this information also needs to be included in the methods section hence we have added these in lines 772-775 in the **Revised MS** and also mentioned in the Figure Legends of respective figures.

"**Figure 1:** **Representative flow cytometer images (BD LSRFortessaTM Cell Analyzer) showing the % of** metacyclics and procyclic LD-promastigote population in the culture of **(A.i.)** GFP-expressing antimony-sensitive (LD-S-GFP) and **(A.ii.)** RFP-expressing antimony-resistant (LD-R-RFP) **after uniform sorting** in Beckman Coulter Cytoflex srt. FSC^{low} (left) represents the metacyclic population and FSC^{high} (right) represents the procyclic population."

"**Figure S1.A**. **Representative flow cytometer images (BD LSRFortessaTM Cell Analyzer) showing the % of** metacyclic promastigote in AG83, BHU777 (7th-day culture); BHU814, BHU575 (5th-day culture) **after uniform sorting** in Beckman Coulter Cytoflex srt. **FSC^{low} (left) represents the metacyclic population and FSC^{high} (right) represents the procyclic population**"

B. Calcein is not a reliable method for LIP quantification in this context (doi: 10.1042/BJ20061840, among others).

Response: This is an important issue and we would like to thank the reviewer for raising this. Firstly, we would like to mention that multiple reports have used Calcein-based assays for the labile iron pool (LIP) monitoring (Prus and Fibach 2008, Cytometry A ; Riedelberger, Penninger 2020, Cell host & microbe). However, we do appreciate the point the reviewer is making here. Please note we have also cited the article that the reviewer has referred to (Tenopoulou, Kurz 2007, The Biochemical journal) in his comment. We would like to bring to the reviewer's attention that this article states that "Calcein fluorescence is quenched following chelation of low-mass labile iron, and the degree of quenching gives an estimate of the amounts of chelatable iron." Furthermore, this paper also points out that, "calcein does not readily chelate iron at lysosomal pH (pH 4–5), the calcein-AM method will not demonstrate all intracellular low-mass iron, but only a fraction of it." Since it is well reported that Lipophosphoglycan of *Leishmania donovani* inhibits phagolysosomal maturation at an early stage of infection to prevent acidification (Vinet, Fukuda 2009, PLoS pathogens ; Verma, Rastogi 2017, PLoS pathogens), we argue that this allows a reliable quantification of LIP by Calcein during early stage of Leishmania infection (at 4hrs pi represented in **Figure 4A.i.**, **A.ii.**, **4B.**, **D.ii.**, and **S3D in the Revised MS and Original MS**).

We would also like to bring to the reviewer's attention that for this particular reason, we have not used calcein-based quantification at the late hours of infection (24hrs pi) when the parasitophorous vacuole becomes acidic due to lysosomal fusion. For late hours thus we have quantified LIP by using another well-accepted method i.e. Ferrozine-based colorimetric assay (Riemer, Hoepken 2004, Anal Biochem ; Banerjee and Datta 2020, Cellular microbiology ; Banerjee and Datta 2023, The Journal of biological chemistry) . It is to be further noted that both these independent quantification methods yielded similar observations at both 4hrs and 24hrs pi (**Figure S2A.i and S2A.ii** in the **Revised and Original MS**) which further validate this methodology and strengthens our claim. Since these multiple quantifications provided similar results, this should essentially remove any doubts regarding the methodology.

C. Many western blot-related conclusions are quantitative, yet the manuscript does not report how these quantifications were performed or the number of replicates.

Response: As we have already mentioned earlier in response to **Comment 5**, we have not included the densitometry analysis in the **Original Submission** for the western blot as the change in band intensities was prominent enough to point out the quantitative differences in most cases, thus we thought including them might overburden the readers. However, as suggested by the reviewer we have now included the Densitometry analysis for all the western blots provided in this MS with their respective statistical analysis represented in **Figure R10 and Figure RS1**). Please note that these Densitometry plots are now presented for respective western blot images in the **Revised MS.**

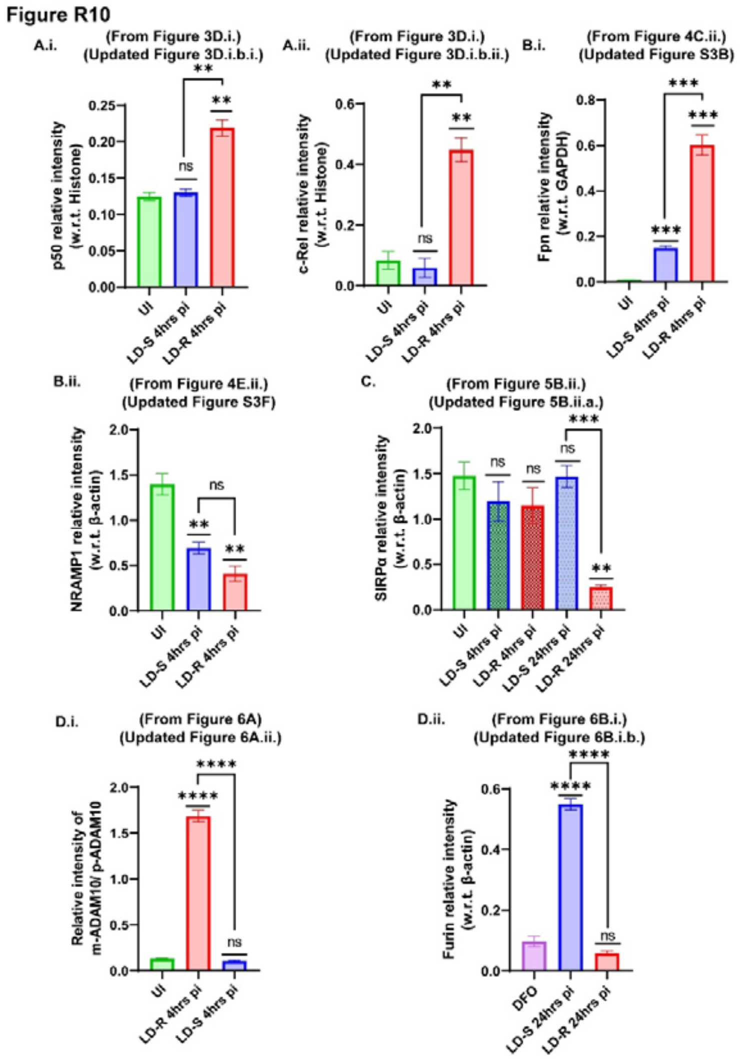

Figure R10: Western blot analysis showing the relative intensity of proteins i.e. fold change with respect to housekeeping control *i.e.* Histone for nuclear fraction, GAPDH and β-actin for whole cell lysate. **(A.i. and A.ii.)** Significant enrichment of nuclear p50 and c-Rel respectively observed in LD-R 4hrs pi as compared to UI and LD-S 4hrs pi ($P \leq 0.01$ marked as **) whereas LD-S 4hrs pi shows no significant change (ns) compared to UI ($P > 0.05$). **(B.i.)** Significant upregulation of Ferroportin (Fpn) in both LD-S and LD-R 4hrs pi (***, $P \leq 0.001$) as compared to UI, with significant upregulation in LD-R 4hrs pi as compared to LD-S 4hrs pi (***, $P \leq 0.001$). **(B.ii.)** Significant downregulation of relative intensity of NRAMP1 in both LD-S and LD-R at 4hrs pi as compared to UI, whereas no significant change is observed in between both these infected experimental conditions. **(C.)** Significant downregulation of relative intensity of SIRPα in LD-R 24hrs pi as compared to UI (**, $P \leq 0.01$) and LD-S 24hrs pi (***, $P \leq 0.001$). **(D.i.)** Graph representing relative intensity analysis of mature-ADAM10 (m-ADAM10) vs precursor-ADAM10 (p-ADAM10). Significant upregulation of relative intensity of m-ADAM10 with respect to p-ADAM10 in LD-R 24hrs pi as compared to both UI and LD-S 24hrs pi (****, $P \leq 0.0001$) whereas no change is observed between LD-R 24hrs pi and UI (ns, $P > 0.05$). **(D.i.)** Significant enrichment of Furin observed in LD-S as compared to both DFO-treated control and LD-R 24hrs pi (****, $P \leq 0.0001$) whereas no change is observed between LD-R 24hrs pi and DFO-treated control (ns, $P > 0.05$). Each densitometry analysis is presented as a bar graph of Mean ± SEM for 3 biological replicates.

Figure RS1

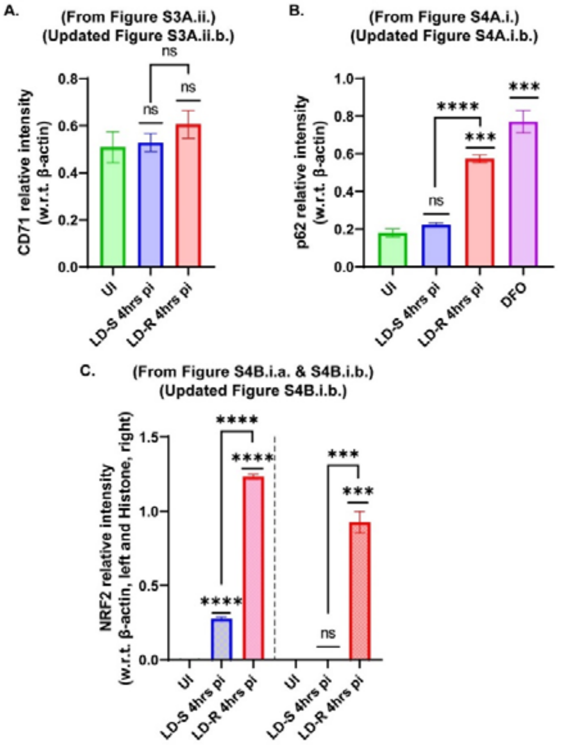

Figure RS1: Western blot analysis showing the relative intensity of proteins i.e. fold change with respect to housekeeping control *i.e.* Histone for nuclear fraction and β-actin for whole cell lysate. **(A.)** No significant change in the relative intensity of CD71 in UI, LD-S 4hrs pi, and LD-R 4hrs pi ($P > 0.05$). **(B.)** Significant upregulation of p62 in both LD-R 4hrs pi and DFO-treated control as compared to UI (***, $P \le 0.001$). Also, significant upregulation in p62 expression in LD-R 4hrs pi as compared to LD-S 4hrs pi (****, $P \le 0.0001$). **(C.)** The relative intensity of NRF2 from whole cell lysate (left panel) with respect to β-actin showed significant upregulation of NRF2 in both LD-S and LD-R infected Mϕs 4hrs pi (****, $P \le 0.0001$). Also, there is significant upregulation in LD-R 4hrs pi vs LD-S 4hrs pi (****, $P \le 0.0001$), significant upregulation of nuclear-NRF2 (right panel) in LD-R 4hrs pi as compared to both UI and LD-S 4hrs pi (***, $P \le 0.001$) is observed. No significant change was observed in the nuclear translocation of NRF-2 with respect to uninfected control for LD-S infection. Each densitometry analysis is represented as a bar graph of Mean ± SEM for 3 biological replicates.

<u>Reviewer #2:</u> The manuscript entitled "Oxidative stress-driven enhanced iron production and scavenging through Ferroportin reorientation worsens anemia in antimony-resistant Leishmania donovani Infection" has investigated possible mechanisms that aid the persistence of Antimony resistant L. donovani parasites, in the Indian subcontinent even after the cessation of antimony treatment in the clinics. These parasites persist better in the host than antimony-sensitive ones. They show that resistant parasites to antimonials scavenge iron more efficiently in the parasitophorous vacuole, than sensitive ones and hence can replicate better as amastigotes. With the use of an antioxidant NAC, they show ROS are required for the survival of the resistant parasites and this was associated with upregulation of OH-1, but this increase was associated with a downmodulation of cytoplasmic Fe+2, contrary to what it was believed. This was associated with an increase of iron, at 4 hr pi of LD-R-Parasitophorous Vacuole. The latter was associated with targeted redistribution of the MΦ membrane iron-exporter, Ferroportin, around LD-R-PV, where the authors say that this causes a downmodulation of cytoplasmic iron and increases the activation of p62/SQSTM1. This is known to degrade the inhibitor of Nrf1 Keap1, facilitating the translocation of Nrf1 to the nucleus, a transcription factor known to initiate the antioxidant response. This iron insufficiency results in increased erythrophagocytosis, via SIRPα degradation, mediated by furin and ADAM10. The authors suggest that erythrophagocytosis is the cause of anaemia, observed in patients infected with LD-R parasites, as it is the case for infected mice with LD-R parasites. This manuscript is novel and has a lot of different experimental procedures and explains contradicting observations in macrophage-Leishmania interactions, like the activation of ROS for the benefit of the parasite. However the presentation is long and complex and the authors need to reassure the readers about the quality of some experiments (described in major comments). Overall, the positive elements of this manuscript make it worth for publication, if the major comments are addressed.

Author Response: We sincerely thank the reviewer for the positive assessment of our work and for appreciating its novelty and the different experimental approaches used to explain contradicting observations.

Comment 1: A lot of "unwanted information" which does not contribute to the final conclusion (ie increased iron pool in PV with lower amounts in cytoplasm which contributes to increase in erythrophagocytosis). I would present the result with the low iron HO-1 upregulation in the supplemental material. I would like to see a simplification and more results in the supplemental figures. For some experiments like the transfection of RAW macrophages with dual reporter luciferase, I would like the authors to comment as it is in general established the macrophages cannot be easily transfected with DNA. Also, as the authors present transcriptomics results provided as a service, but there is no clear mention of the quality of these data in the main MS (number of replicas etc). Also, many of these factors that are upregulated or downmodulated, apart from the Iron pathway affect the whole macrophage function (ie HO-1 causes a M2 phenotype in macrophages and hence allows the growth of intracellular parasites). In the discussion, it would be great to see some description between these results and the macrophage programs (inflammatory M1, anti-inflammatory M2).

Author Response:
The reviewer has raised a few concerns which we would like to address each of them in a pointwise manner:

A. A lot of "unwanted information" which does not contribute to the final conclusion (ie increased iron pool in PV with lower amounts in cytoplasm which contributes to increase in erythrophagocytosis). I would present the result with the low iron HO-1 upregulation in the supplemental material.

Response: We agree with this assessment of the reviewer. We have added much information and additional experimental approaches to the main figures with the intention of validating every observation with additional approaches. However, we do feel that as a consequence, the figures have become much dense. Hence, as per the reviewer's suggestion, we have updated the main figures in the revised manuscript and transfer additional data (like the result of low iron) to the supplementary section to make it as simple and crisp and reduce the data load. Please note we however have to include the Densitometry analysis in few main figures of the **Revised MS** as this was specifically requested by reviewer1.

B. For some experiments like the transfection of RAW macrophages with dual reporter luciferase, I would like the authors to comment as it is in general established the macrophages cannot be easily transfected with DNA.

Response: For the Dual-Luciferase assay, as correctly pointed out by the reviewer it's very difficult to achieve significant transfection in primary macrophages. Hence, we have used RAW 264.7 macrophage cell line which has been previously well-reported for achieving successful transfection using lipofectamine (Mukherjee, Mukhopadhyay 2013, Proceedings of the National Academy of Sciences of the United States of America ; Kang, Kwon 2014, Gene Ther) and we also observed the same result. We have mentioned this in the Materials and Methods section **of the Revised MS** in line 710-711 as "For difficulty in achieving high efficiency transfection in primary murine macrophages, RAW 264.7 macrophage cell lines were transiently transfected with HO1+PGL3, SiteA$^{-/-}$HO1+PGL3, and SiteB$^{-/-}$HO1+PGL3 plasmids using Lipofectamine 2000 reagent (Invitrogen) for 6hrs, washed and incubated for another 12hrs followed by infection with LD-S or LD-R or kept uninfected".

C. Also, as the authors present transcriptomics results provided as a service, but there is no clear mention of the quality of these data in the main MS (number of replicas etc).

Response: We agree and we have elaborated on the transcriptomics results in the **Revised MS**. We have attached the screenshot of the QC report indicating the quality of this data for your reference and the QC table as follows:

Library QC Report

**Kit used for Extraction**	NEBNext® Ultra™ II Directional RNA Library Prep Kit for Illumina
**Library QC Details**	Qubit 4.0 fluorometer(Thermofisher #Q33238)<br>Tapestation 2200 (Agilent)
**Sequencing Chemistry**	150XPE
**Sequencing Platform**	Nova Seq 6000
**Conclusion**	Pass
**Remarks**	Remarks

Library QC table

Sl	Sample Name	Qubit ng/ul	QC Status	Volume (µL)
1	UI-1	11.2	PASS	15
2	UI-2	9.6	PASS	15
3	LD-S 4hrs pi-1	16.3	PASS	15
4	LD-S 4hrs pi-2	14.3	PASS	15
5	LD-R 4hrs pi-1	6.59	PASS	15
6	LD-R 4hrs pi-2	11.9	PASS	15
7	LD-S 24hrs pi-1	5.36	PASS	15
8	LD-S 24hrs pi-2	7.96	PASS	15
9	LD-R 24hrs pi-1	12.6	PASS	15
10	LD-R 24hrs pi-2	4.74	PASS	15

The number of replicates used in this study is two. In brief, we have used the average normalized counts of the replicates using DESeq2 R packages for producing the heatmap. We have updated this information in the RNAseq materials and methods (lines 660-662) in the **Revised MS** as follows:

"Normalized counts (N=2) were obtained from the DESeq2 package and averaged normalized counts were used to develop heatmaps of a few selected genes using gplots and RColorBrewer."

D. Also, many of these factors that are upregulated or downmodulated, apart from the Iron pathway affect the whole macrophage function (ie HO-1 causes a M2 phenotype in macrophages and hence allows the growth of intracellular parasites). In the discussion, it would be great to see some description between these results and the macrophage programs (inflammatory M1, anti-inflammatory M2).

Response: We appreciate the reviewer's insightful suggestion and agree that it would be useful to look into factors apart from the iron pathway that are significantly upregulated or downregulated affecting the overall macrophage programming and eventually promoting the aggressive growth of intracellular-resistant parasites. As per the reviewer's suggestion, we have analyzed our RNAseq data to dig into the inclination of the differential polarity of macrophages for LD-S vs LD-R infected macrophages at 4hrs and 24hrs pi which might contribute to the aggressive proliferation of LD-R and have added this as **Figure S2B.ii.** in the **Revised Supplementary section**. We have provided a brief paragraph describing the final outcome of polarization of macrophages in the methodology section lines 662-671 as "To decode the differential polarity of MΦs infected with LD-S vs LD-R at 4hrs and 24hrs pi, the gene sets contributing towards M1 and M2 phenotypes were obtained from the previous report (71) and tallied with our RNAseq data. Score of M1/ M2 gene cluster = Sum of respective M1/M2 $\log_2$(fold change) of genes with adjusted *p-value*<0.05.

The difference in these scores indicates the inclination of the polarity of MΦs toward M1 or M2 polarization (-ve indicating M2 polarization and +ve indicating M1 polarization).

$$Score\ of\ M1/M2\ polarization\ gene\ set = Sum\ of\ resepective\ Log2FoldChange\ of\ genes\ with\ adj\ p-value < 0.05$$

$Final\ determinant\ score\ = Score\ from\ M1\ gene\ Set - Score\ from\ M2\ gene\ Set$". And in the results section lines 274-280 as "At 4hrs pi, both LD-S and LD-R infection showed an inclination towards M1 phenotype, with LD-R showing a higher degree of M1 polarization state possibly due to higher ROS induction **(Figure S2B.ii., and Supplementary Excel Sheet 1)**. However, at later time points, LD-R infections showed a complete shift towards the M2 phenotype. This huge shift towards the M2 phenotype might help LD-R to survive and proliferate more inside the host as compared to LD-S which showed relatively less inclination towards the M2 phenotype **(Figure S2B.ii.**, and **Supplementary Excel Sheet 1)**." of the **Revised MS**. We have also provided the list of genes contributing towards the macrophage polarization in **Supplementary Excel Sheet 1**.

Part III – Minor Issues: Editorial and Data Presentation Modifications
Please use this section for editorial suggestions as well as relatively minor modifications of existing data that would enhance clarity.

Reviewer #1: (No Response)

Reviewer #2: Some typos and grammatical errors. Ie line 407 intracellular proliferation (requires a full stop). I would also like to see an abbreviation list. Also, in the results section it is not always easy to understand if some experiments are performed in promastigotes, amastigotes or macrophages.

Author Response: We thank the reviewer for pointing out this typo in line 407 which we somehow overlooked. We have changed this in the **Revised manuscript** line number 434. We have further look into the whole manuscript to fix any sort of typos and grammatical errors in the **Revised manuscript**.

REFERENCES:

1. de Paiva RM, Grazielle-Silva V, Cardoso MS, Nakagaki BN, Mendonca-Neto RP, Canavaci AM, et al. Amastin Knockdown in Leishmania braziliensis Affects Parasite-Macrophage Interaction and Results in Impaired Viability of Intracellular Amastigotes. PLoS pathogens. 2015;11(12):e1005296.
2. Roy M, Sarkar D, Chatterjee M. Quantitative monitoring of experimental and human leishmaniasis employing amastigote-specific genes. Parasitology. 2022;149(8):1085-93.
3. Khanra S, Sarraf NR, Das AK, Roy S, Manna M. Miltefosine Resistant Field Isolate From Indian Kala-Azar Patient Shows Similar Phenotype in Experimental Infection. Scientific reports. 2017;7(1):10330.
4. Mukherjee S, Mukherjee B, Mukhopadhyay R, Naskar K, Sundar S, Dujardin JC, et al. Imipramine is an orally active drug against both antimony sensitive and resistant Leishmania donovani clinical isolates in experimental infection. PLoS neglected tropical diseases. 2012;6(12):e1987.
5. Cotterell SE, Engwerda CR, Kaye PM. Enhanced hematopoietic activity accompanies parasite expansion in the spleen and bone marrow of mice infected with Leishmania donovani. Infection and immunity. 2000;68(4):1840-8.
6. Lang T, Ave P, Huerre M, Milon G, Antoine JC. Macrophage subsets harbouring Leishmania donovani in spleens of infected BALB/c mice: localization and characterization. Cellular microbiology. 2000;2(5):415-30.
7. Srivastav S, Basu Ball W, Gupta P, Giri J, Ukil A, Das PK. Leishmania donovani prevents oxidative burst-mediated apoptosis of host macrophages through selective induction of suppressors of cytokine signaling (SOCS) proteins. The Journal of biological chemistry. 2014;289(2):1092-105.
8. Ouakad M, Vanaerschot M, Rijal S, Sundar S, Speybroeck N, Kestens L, et al. Increased metacyclogenesis of antimony-resistant Leishmania donovani clinical lines. Parasitology. 2011;138(11):1392-9.
9. Prus E, Fibach E. Flow cytometry measurement of the labile iron pool in human hematopoietic cells. Cytometry A. 2008;73(1):22-7.
10. Riedelberger M, Penninger P, Tscherner M, Seifert M, Jenull S, Brunnhofer C, et al. Type I Interferon Response Dysregulates Host Iron Homeostasis and Enhances Candida glabrata Infection. Cell host & microbe. 2020;27(3):454-66 e8.
11. Tenopoulou M, Kurz T, Doulias PT, Galaris D, Brunk UT. Does the calcein-AM method assay the total cellular 'labile iron pool' or only a fraction of it? The Biochemical journal. 2007;403(2):261-6.
12. Vinet AF, Fukuda M, Turco SJ, Descoteaux A. The Leishmania donovani lipophosphoglycan excludes the vesicular proton-ATPase from phagosomes by impairing the recruitment of synaptotagmin V. PLoS pathogens. 2009;5(10):e1000628.
13. Verma JK, Rastogi R, Mukhopadhyay A. Leishmania donovani resides in modified early endosomes by upregulating Rab5a expression via the downregulation of miR-494. PLoS pathogens. 2017;13(6):e1006459.
14. Riemer J, Hoepken HH, Czerwinska H, Robinson SR, Dringen R. Colorimetric ferrozine-based assay for the quantitation of iron in cultured cells. Anal Biochem. 2004;331(2):370-5.
15. Banerjee S, Datta R. Leishmania infection triggers hepcidin-mediated proteasomal degradation of Nramp1 to increase phagolysosomal iron availability. Cellular microbiology. 2020;22(12):e13253.
16. Banerjee S, Datta R. Localized Leishmania major infection disrupts systemic iron homeostasis that can be controlled by oral iron supplementation. The Journal of biological chemistry. 2023;299(8):105064.
17. Mukherjee B, Mukhopadhyay R, Bannerjee B, Chowdhury S, Mukherjee S, Naskar K, et al. Antimony-resistant but not antimony-sensitive Leishmania donovani up-regulates host IL-10 to overexpress multidrug-resistant protein 1. Proceedings of the National Academy of Sciences of the United States of America. 2013;110(7):E575-82.
18. Kang WS, Kwon JS, Kim HB, Jeong HY, Kang HJ, Jeong MH, et al. A macrophage-specific synthetic promoter for therapeutic application of adiponectin. Gene Ther. 2014;21(4):353-62.
